# Assembly and characterisation of a unique onion diversity set identifies resistance to Fusarium basal rot and improved seedling vigour

**DOI:** 10.1007/s00122-019-03422-0

**Published:** 2019-09-13

**Authors:** Andrew Taylor, Graham R. Teakle, Peter G. Walley, William E. Finch-Savage, Alison C. Jackson, Julie E. Jones, Paul Hand, Brian Thomas, Michael J. Havey, David A. C. Pink, John P. Clarkson

**Affiliations:** 1 Warwick Crop Centre, School of Life Sciences, University of Warwick, Wellesbourne Campus, Wellesbourne, Warwick, CV35 9EF UK; 2 Institute of Integrative Biology, University of Liverpool, Liverpool, L69 3BX UK; 3 Crop and Environment Sciences, Harper Adams University, Newport, T10 8NB UK; 4 School of Life Sciences, University of Warwick, Coventry, CV4 7AL UK; 5 USDA-ARS, Department of Horticulture, University of Wisconsin, 1575 Linden Drive, Madison, WI 53706 USA

## Abstract

**Key message:**

A unique, global onion diversity set was assembled, genotyped and phenotyped for beneficial traits. Accessions with strong basal rot resistance and increased seedling vigour were identified along with associated markers.

**Abstract:**

Conserving biodiversity is critical for safeguarding future crop production. Onion (*Allium cepa* L.) is a globally important crop with a very large (16 Gb per 1C) genome which has not been sequenced. While onions are self-fertile, they suffer from severe inbreeding depression and as such are highly heterozygous as a result of out-crossing. Bulb formation is driven by daylength, and accessions are adapted to the local photoperiod. Onion seed is often directly sown in the field, and hence seedling establishment is a critical trait for production. Furthermore, onion yield losses regularly occur worldwide due to Fusarium basal rot caused by *Fusarium oxysporum* f. sp. *cepae*. A globally relevant onion diversity set, consisting of 10 half-sib families for each of 95 accessions, was assembled and genotyping carried out using 892 SNP markers. A moderate level of heterozygosity (30–35%) was observed, reflecting the outbreeding nature of the crop. Using inferred phylogenies, population structure and principal component analyses, most accessions grouped according to local daylength. A high level of intra-accession diversity was observed, but this was less than inter-accession diversity. Accessions with strong basal rot resistance and increased seedling vigour were identified along with associated markers, confirming the utility of the diversity set for discovering beneficial traits. The onion diversity set and associated trait data therefore provide a valuable resource for future germplasm selection and onion breeding.

**Electronic supplementary material:**

The online version of this article (10.1007/s00122-019-03422-0) contains supplementary material, which is available to authorized users.

## Introduction

Onion (*Allium cepa* L.) is a major horticultural crop belonging to the order Asparagales which, of the monocot orders, is second only to the Poales for global economic value (Kuhl et al. 2004). Worldwide, 93 Mt of bulb onions are produced annually (FAOSTAT 2016) and onion consumption is associated with potential health benefits, mainly linked to alk(en)yl cysteine sulphoxides and flavonoid compounds (Griffiths et al. 2002). Although onions are self-fertile, they are considered outbreeding crops as they exhibit severe inbreeding depression (Jones and Davis 1944; Brewster 2008). In one study, 75% of seed derived from cross-pollination produced bulbs in a field trial, whereas less than 50% of seed from self-pollination produced bulbs (Currah and Ockendon 1983). This means that most onion cultivars are heterozygous with each plant being genetically unique. Onion is a diploid species (2*n* = 2*x* = 16) but possesses one of the largest genomes among eukaryotes (16.3 Gb per 1C nucleus; Arumuganathan and Earle 1991). Together, the large genome and outbreeding nature of onion have significantly hampered the development of advanced genomic resources compared to most other major crops. Although there is no publicly available nuclear DNA sequence, both the mitochondrial and chloroplast genomes have been sequenced (Shigyo et al. 2018). Molecular markers for genotyping have been developed through restriction fragment length polymorphisms (RFLPs), amplified fragment length polymorphisms (AFLPs), simple sequence repeats (SSRs) and single nucleotide polymorphisms (SNPs) (King et al. 1998; van Heusden et al. 2000; Martin et al. 2005; Baldwin et al. 2012a, b; Jo et al. 2017). More recently, Duangjit et al. (2013) reported the development of 930 validated Kompetitive Allele-Specific PCR (KASP™; developed by LGC Ltd.) SNP assays from transcriptome sequencing, together with a reference genetic map.

Daylength sensitivity is a key trait for onion production. In the UK and northern Europe, long-day (LD) cultivars requiring > 16 h day light for bulb initiation are grown, while in other parts of the world, intermediate day (ID; requiring 13–14 h daylength) or short-day (SD; requiring 12 h daylength) cultivars are grown (Brewster 2008). Therefore, breeding programmes are generally confined to germplasm adapted to each specific daylength, generating a major breeding bottleneck for the exchange of alleles between the different daylength-adapted genepools. There is also considerable variability between onion cultivars for important traits such as bulb colour/size, time to maturity and pungency suggesting that genetic diversity within *A. cepa* exists (Griffiths et al. 2002). Given the increasing speed of genetic erosion in *A. cepa* and related species (Keller et al. 2013), genebanks play a crucial role in the conservation of genetic diversity so that it can be further exploited to discover alleles for beneficial traits. A good example of the value of a closely related species is the introgression of downy mildew resistance into *A. cepa* from *A. roylei* (Scholten et al. 2007). However, the difficulty in introgressing this resistance without other associated unfavourable alleles meant that it took approximately 20 years to breed this trait into a commercial onion cultivar (Scholten et al. 2007). Therefore, to shorten the breeding process, it is more efficient to identify beneficial alleles within *A. cepa* germplasm.

For much of onion production, seed is directly sown in the field and hence seedling establishment is an important trait for a uniform, high yielding crop (Finch-Savage et al. 2010). Seedling emergence has a direct effect on crop yield and cannot be compensated for with subsequent inputs (Bleasdale 1967), while predictable and even emergence is crucial for crop uniformity and for effective timing of herbicide applications, required to maximise yield. Seedling vigour is an indicator of how well a seed lot will establish seedlings (Finch-Savage et al. 2010; Finch-Savage and Bassel 2016) and is determined by the interaction between genetics and environment (Whittington 1973; Hodgkin and Hegarty 1978). It is important to consider both germination and post-germination seedling growth. Three main traits have been established for measuring vigour: germination speed, speed of initial downward root growth and upward shoot growth in compacted soil (Finch-Savage et al. 2010). However, the standard test used to measure seed quality in the onion industry is a simple filter paper germination test (ISTA 2013). While this can be used to measure viability, it gives little information on vigour. Effective tests have been developed to measure all three of the main seedling vigour traits in *Brassica oleracea* under controlled conditions (Bettey et al. 2000; Finch-Savage et al. 2010).

One of the major constraints to onion production worldwide is Fusarium basal rot (FBR) caused by the soilborne fungus *Fusarium oxysporum* f. sp. *cepae* (FOC). The pathogen can cause symptoms at all stages of plant development leading to both pre- and post-harvest losses (Entwistle 1990; Taylor et al. 2013). FOC is a major problem for onion growers in the UK, and losses are predicted to increase further under current climate change models as infection is favoured by warmer temperatures (optimum 30 °C; Cramer 2000). As FOC produces chlamydospores that can survive in the soil for many years, disease management is challenging and has previously relied on soil sterilisation or fungicides (Brayford 1996; Cramer 2000). These approaches have largely been unsuccessful, have undesirable environmental effects and are threatened restrictions on pesticide use. The development of resistant cultivars is therefore a desirable option. Onion seedling assays have been employed for rapid initial screening for FBR resistance (Retig et al. 1970; Abawi and Lorbeer 1971b; Holz and Knox-Davies 1974; Krueger et al. 1989; Galván et al. 2008; Saxena and Cramer 2009; Taylor et al. 2013) and using this approach together with a subsequent mature plant assay, partial resistance has been identified (Taylor et al. 2013). This supports previous reports where only partial resistance to FBR has ever been identified in onion (Cramer 2000; Saxena and Cramer 2009; Gei et al. 2014). High-level resistance has been reported in closely related *Allium* species such as *A. roylei*, *A. fistulosum* and *A. galanthum* (Galván et al. 2008; Rout et al. 2016), but these species do not produce a bulb and would require a long breeding programme to achieve a commercial FBR resistant onion cultivar. FOC isolates can also vary in their aggressiveness (Özer et al. 2004; Galván et al. 2008; Taylor et al. 2013), although there does not always appear to be a FOC isolate × cultivar interaction (Taylor et al. 2013). The best strategy is therefore to identify and utilise a highly aggressive FOC isolate for FBR resistance screening.

Here we report the development of a unique onion diversity set consisting of up to 10 half-sib families (Cramer 2006) for each of 95 accessions to enable pre-breeding (trait screening) research. Genotyping using 892 SNP markers was utilised to determine the underlying population structure and relatedness. The utility of the diversity set was also assessed by screening one half-sib family per accession for response to FOC and improved seedling vigour leading to the identification of accessions with high levels of basal rot resistance and increased vigour. These phenotype data were then used to explore marker-trait associations.

## Materials and methods

### Development of an onion diversity set

Ninety-six onion accessions were selected from the UK Vegetable Genebank, University of Warwick (Table 1) considering geographic origin (including LD, ID and SD types) and including a mixture of advanced cultivars, traditional cultivars and landraces. *A. cepa var. ascalonicum* (shallot), *A. fistulosum* (bunching onion) *A. cepa* × *A. fistulosum* cross and the two wild species *A. roylei* and *A. vavilovii* were also included. Seed was sown in beds in Wellesbourne, UK in April 2006. Bulbs were harvested at maturity, dried and in late 2006–early 2007, ten healthy bulbs per accession planted in pots in separate insect proof cages in a glasshouse under natural conditions. Upon flowering, blow flies were used to randomly cross-pollinate or self-pollinate the ten plants and seed was collected from each plant individually. The seed from each plant therefore represents a half-sib (HS) family for which the female parent is known, but the male parent could be any one of the ten plants from the same accession. Seed was stored under Genebank long-term storage conditions (− 20 C, 5% moisture content; FAO 2013) in hermetically sealed foil-laminate pouches.

**Table 1:** Onion diversity set accessions including the number of half-sib (HS) families created from each founder line and percentage heterozygosity for one mother plant of a single representative HS family from each accession

Genus/species	Accession name	Code	Accession no.	Common name	Origin	Daylength response	No. of HS families	% Het	Phenotyping
A. cepa	AGRIFOUND ROSE	AR	HRIGRU009799	Bulb onion	India	**SD**	6	26.3	FS
A. cepa	AILSA CRAIG	AC	HRIGRU011280	Bulb onion	UK	**LD**	10	29.6	FS
A. cepa var. ascalonicum	AMBITION (F1)	AF	Lot E057391	Hybrid shallot	Netherlands	n/a	10	38.5	FS
A. cepa	AUXONNE	AU	HRIGRU004648	Bulb onion	France	LD	10	38.7	FS
A. cepa	BABOSA JENAN	BJ	HRIGRU007010	Bulb onion	Japan	SD	8	26.3	FS
A. cepa	BALAKLEEVSKIJ	BA	HRIGRU004639	Bulb onion	Russia	**LD**	10	34.5	FS
A. cepa	BARLETTA	BR	HRIGRU006417	Bulb onion	Israel	SD	10	28.4	FS
A. cepa	BEDFORDSHIRE CHAMPION	BC	HRIGRU000130	Bulb onion	UK	**LD**	10	33.0	FS
A. cepa	BEN SHEMEN	BS	HRIGRU005772	Bulb onion	Israel	SD	8	31.2	FS
A. cepa	BETH ALPHA AUTUMN	BE	HRIGRU006285	Bulb onion	Israel	SD	7	4.9	S
A. cepa	BLOOD RED BRUNSWICK	BB	HRIGRU002908	Bulb onion	UK	**LD**	10	34.1	FS
A. cepa	BRAESIDE GOLDEN GLOBE	BG	HRIGRU000230	Bulb onion	Australia	ID	10	30.9	FS
A. cepa	BROWN SPANISH	BRS	HRIGRU000089	Bulb onion	Spain	ID	10	33.6	FS
A. cepa	BUAN	BU	HRIGRU005070	Bulb onion	Ireland	**LD**	10	31.2	FS
A. cepa	BUFFALO (F1)	BF	HRIGRU004151	Hybrid bulb onion	Netherlands	LD/ID	7	34.3	S
A. cepa	CALIFORNIAN RED	CR	HRIGRU005548	Bulb onion	New Zealand	**SD**	9	21.3	FS
A. cepa	CANDY (F1)	CA	HRIGRU013760	Hybrid bulb onion	USA	**ID**	10	35.2	FS
A. cepa	CEBOLA BRANCA	CB	HRIGRU009581	Bulb onion	Portugal	ID	10	35.5	FS
A. cepa	CEBOLA VERMELHA	CV	HRIGRU009583	Bulb onion	Portugal	LD/ID	10	30.6	FS
A. cepa	CEBOLLA VALENCIANA	CVA	HRIGRU009862	Bulb onion	Spain	LD	9	25.4	FS
A. cepa	CIPOLLINA	CI	HRIGRU005464	Bulb onion	Italy	?	2	nd	None
A. cepa	CREAMGOLD	CG	HRIGRU005640	Bulb onion	Australia	ID	10	36.5	FS
A. cepa	DORATA DI PALMA	DP	HRIGRU005440	Bulb onion	Italy	LD	10	29.9	FS
A. cepa	DOWNING'S YELLOW GLOBE	DY	HRIGRU006634	Bulb onion	USA	**LD**	10	38.0	FS
A. cepa	DOWNY MILDEW RESISTANT	DM	HRIGRU006326	Bulb onion	Spain	**LD**	10	5.8	FS
A. cepa	EARLY RED	ER	HRIGRU010801	Bulb onion	Israel	**SD**	10	27.2	FS
A. cepa	EXCEL	EX	HRIGRU012469	Bulb onion	USA	**SD**	7	21.6	FS
A. cepa	EXHIBITION	EH	HRIGRU005512	Bulb onion	UK	**LD**	10	36.5	FS
A. cepa	FIESTA (F1)	FI	HRIGRU008423	Hybrid bulb onion	USA	LD/ID	10	30.4	FS
A. cepa	GELBE WIENER	GW	HRIGRU006397	Bulb onion	Austria	**LD**	10	32.8	FS
A. cepa	GIANT ROCCA BROWN	GR	HRIGRU006001	Bulb onion	Italy	LD/ID	10	29.6	FS
A. cepa	GIANT ZITTAU	GZ	HRIGRU000156	Bulb onion	UK	**LD**	10	32.0	FS
A. cepa	GIZA 20	GI	HRIGRU004221	Bulb onion	Egypt	**SD**	7	13.3	None
A. cepa	GLADALAN BROWN	GB	HRIGRU000235	Bulb onion	Australia	ID	8	28.5	S
A. cepa	GRANOBLE	GN	HRIGRU010806	Bulb onion	USA	SD	8	24.0	FS
A. cepa	GREENELLA	GRE	HRIGRU012891	Bulb onion	Germany	**LD**	10	22.2	FS
A. cepa × A. fistulosum	GUARDSMAN	GU	HRIGRU011176	Bunching onion	UK	LD	10	33.3	FS
A. fistulosum	HARDY LONG WHITE	HLW	HRIGRU000121	Bunching onion	unknown	n/a	7	0.5	FS
A. cepa	HIBERNA	HI	HRIGRU004093	Bulb onion	Czech Republic	**LD**	10	33.9	FS
A. cepa	HOJEM	HO	HRIGRU001825	Bulb onion	South Africa	**SD**	10	35.8	FS
A. cepa	HYDURO (F1)	HYD	HRIGRU006006	Hybrid bulb onion	Netherlands	**LD**	10	34.2	FS
A. cepa	HYSOL (F1)	HYS	HRIGRU006037	Hybrid bulb onion	Netherlands	**LD**	10	36.6	FS
A. cepa	IMAI EARLY YELLOW	IY	HRIGRU006075	Bulb onion	Japan	**ID**	8	30.2	FS
A. cepa	JAUNE DE VALENCE	JV	HRIGRU010813	Bulb onion	France	**LD**	10	31.0	FS
A. cepa	JAUNE DES CEVENNES	JC	HRIGRU012917	Bulb onion	France	**LD**	10	34.3	FS
A. cepa	KUTNOWSKA	KU	HRIGRU000040	Bulb onion	Poland	**LD**	10	37.0	FS
A. cepa	LAND RACE (from Russia)	LR	HRIGRU002427	Bulb onion	Russia	**LD**	10	35.2	FS
A. cepa	LJASKOVSKI 58	LJ	HRIGRU000079	Bulb onion	Bulgaria	LD	10	33.0	FS
A. cepa	MAKOI BRONZ	MB	HRIGRU009858	Bulb onion	Hungary	**LD**	10	30.9	FS
A. cepa	MAKOVSKI	MA	HRIGRU000083	Bulb onion	Bulgaria	LD	10	36.7	FS
A. cepa	MALAKOFF	MK	HRIGRU000182	Bulb onion	Spain	LD	10	30.0	FS
A. cepa	MITZRI HAEMEK	MH	HRIGRU006416	Bulb onion	Israel	SD/ID	9	32.7	S
A. cepa	MORADA DE AMPOSTA	MDA	HRIGRU009863	Bulb onion	Spain	**ID**	10	26.5	FS
A. cepa	MORAVANKA	MO	HRIGRU005519	Bulb onion	Czech Republic	LD	10	22.0	FS
A. cepa	NEW MEXICO YELLOW GRANO	NM	HRIGRU006122	Bulb onion	USA	**SD**	7	32.8	None
A. cepa	NUMEX BR-1	NU	HRIGRU010804	Bulb onion	USA	**SD**	8	20.7	FS
A. cepa	ODOURLESS	OD	HRIGRU000237	Bulb onion	Australia	ID	3	nd	None
A. cepa	OWA	OW	HRIGRU005953	Bulb onion	Denmark	**LD**	10	31.3	FS
A. cepa	POMPEI	PO	HRIGRU006414	Bulb onion	Israel	**SD**	9	31.4	FS
A. cepa	PUKEKOHE LONGKEEPER	PL	HRIGRU005524	Bulb onion	New Zealand	**LD**	10	38.4	FS
A. cepa	PUSA RED	PR	HRIGRU009798	Bulb onion	India	**SD**	3	nd	None
A. cepa	RADAR	RA	HRIGRU013815	Bulb onion	UK	**LD**	10	39.1	FS
A. cepa	RAWSKA	RAW	HRIGRU000183	Bulb onion	Poland	**LD**	10	31.9	FS
A. cepa	RED SYNTHETIC	RS	HRIGRU010800	Bulb onion	Israel	**SD**	7	18.6	FS
A. cepa	RED TORPEDO	RT	HRIGRU010609	Bulb onion	Italy	ID	10	24.0	FS
A. cepa	RED WETHERFIELD	RW	HRIGRU006017	Bulb onion	Netherlands	**LD**	10	36.8	FS
A. cepa	RIJNSBURGER BALSTORA	RB	HRIGRU004130	Bulb onion	UK	**LD**	10	34.1	FS
A. cepa	RIJNSBURGER JUMBO	RJ	HRIGRU004200	Bulb onion	UK	**LD**	10	30.1	FS
A. cepa	ROSE DE ROSCOFF	RR	HRIGRU006746	Bulb onion	France	LD	10	40.4	FS
A. cepa	ROSSA DI FIRENZE	RF	HRIGRU000194	Bulb onion	Italy	LD	10	31.7	FS
A. cepa	S R ROND	SRR	HRIGRU006742	Bulb onion	France	LD	10	29.3	FS
A. cepa	SAJOVAMOS	SA	HRIGRU005766	Bulb onion	Hungary	**LD**	10	34.6	FS
A. cepa	SAPPORO KI	SK	HRIGRU007812	Bulb onion	Japan	**LD**	9	36.2	FS
A. cepa	SARAND	SR	HRIGRU005769	Bulb onion	Hungary	LD	10	38.3	FS
A. cepa	SENSHYU YELLOW	SY	HRIGRU006077	Bulb onion	Japan	**ID**	9	34.1	FS
A. cepa	SHAKESPEARE	SH	HRIGRU012628	Bulb onion	UK	**LD**	10	23.3	FS
A. cepa	SOUTHPORT WHITE GLOBE	SW	HRIGRU006631	Bulb onion	USA	**LD**	10	34.0	FS
A. cepa	SPEARWOOD	SP	HRIGRU005771	Bulb onion	New Zealand	?	10	31.9	FS
A. cepa	STURON	ST	HRIGRU006036	Bulb onion	Netherlands	**LD**	10	27.5	FS
A. cepa	STUTTGART GIANT	SG	HRIGRU005928	Bulb onion	UK	**LD**	10	37.7	FS
A. cepa	STUTTGARTER RIESEN	STR	HRIGRU006058	Bulb onion	Denmark	**LD**	10	32.9	FS
A. cepa	SWEET ONION	SO	HRIGRU011760	Bulb onion	Italy	?	10	35.6	FS
A. cepa	TEXAS EARLY GRANO 502	TG	HRIGRU005123	Bulb onion	USA	SD	0	nd	None
A. cepa	THE KELSAE	TK	HRIGRU006997	Bulb onion	UK	**LD**	10	25.4	FS
A. cepa	VEGA (F1)	VE	HRIGRU011757	Hybrid bulb onion	Italy	LD/ID	10	40.3	FS
A. cepa	WALLA WALLA SWEET ONION	WW	HRIGRU011194	Bulb onion	USA	**LD**	10	36.4	FS
A. cepa	WHITE CREOLE PRR PVP	WC	HRIGRU011964	Bulb onion	USA	**SD**	10	34.0	FS
A. cepa	WHITE EBENEZER	WE	HRIGRU006018	Bulb onion	Netherlands	**LD**	10	41.6	FS
A. cepa	WHITE LISBON	WL	HRIGRU004092	Bulb onion	UK	**LD**	10	32.6	FS
A. cepa	WHITE SWEET SPANISH JUMBO	WS	HRIGRU005124	Bulb onion	USA	**LD**	10	23.9	FS
A. vavilovii^a^	WILD SPECIES	WSV	HRIGRU002441	Bulb onion	unknown	n/a	10	11.1	FS
A. roylei	WILD SPECIES	WSR	AC021171	Bulb onion	unknown	n/a	10	0.3	F^b^
A. cepa	WOLSKA HOSER	WH	HRIGRU005520	Bulb onion	Poland	**LD**	10	36.8	FS
A. cepa	YELLOW BERMUDA	YB	HRIGRU006715	Bulb onion	USA	**SD**	7	14.3	S
A. cepa	YELLOW SWEET SPANISH UTAH JUMBO	YS	HRIGRU006656	Bulb onion	USA	**LD**	10	34.7	FS
A. cepa	YODALEF	YO	HRIGRU006415	Bulb onion	Israel	**SD**	6	nd	None
*Additional lines used (not belonging to diversity set)*									
A. cepa	HYSTAR	HY		Hybrid bulb onion	Netherlands	**LD**	n/a	47.3	F
A. cepa	SERRANA	SE	HRIGRU13146	Bulb onion	USA	**SD**	n/a	40.8	F
A. cepa	AILSA CRAIG PRIZEWINNER	ACP		Bulb onion	UK	**LD**	n/a	27.6	F

Accession number refers to the UK Vegetable Genebank and all seed was sourced from the Warwick Crop Centre Genetic Resources Unit. Daylength response (SD, short day; LD, long day; ID, intermediate; n/a, not applicable; ?, not known). Bold text indicates that a clear daylength response was derived from how the accessions behaved in trials, geographic origin and any available information from breeders or the Genebank. Accessions were phenotyped for Fusarium resistance (F), seedling vigour (S) or both (FS) depending on the amount of seed available

^a^SNP marker data suggests that this accession has been misidentified and is *A. cepa*

^b^Not fully replicated

### Onion SNP genotyping and analysis

To examine genetic diversity across founder onion accessions and between and within half-sib families, genotyping was carried out using DNA extracted from leaf tissue of (1) one of the 10 HS mother plants from 91 founder onion accessions and three cultivars used as resistant (SE, Saxena and Cramer 2009; ACP, Taylor et al. 2013), or susceptible (HY, Taylor et al. 2013) controls in FOC resistance screens, (2) the nine remaining HS mother plants for four selected onion accessions (CA, DM, GR and HO) and (3) ten individual plants from the same single HS family (same mother plant) for each of the four selected accessions in (2). Leaf tissue was flash frozen in liquid nitrogen, lyophilised and 20 mg disrupted in a lysing matrix A tube (MPBio) by a FastPrep-24™ machine (MPBio) set at 6 ms^−1^ for 40 s. DNA was extracted using a DNeasy plant mini kit (Qiagen), quality checked using a Nanodrop spectrophotometer (Thermo Scientific) and genotyping carried out by LGC Ltd., UK using 892 published KASP™ SNP markers (Duangjit et al. 2013).

Markers were removed from further analysis if they were monomorphic, had a minor allele frequency below 5%, had more than five missing data points or repeatedly gave illegitimate genotypes in the male parent analysis described below. The heterozygosity of each of the 94 HS mother plants was determined using the remaining 765 markers. To examine the genotypic diversity across the 94 representative mother plants from each of the founder accessions, genotype scores were converted to standard IUPAC single letter nucleotide ambiguity codes and used to construct a neighbour-joining (NJ) tree using MEGA 7 (Kumar et al. 2016) with 1000 bootstrap replications. Default parameters were used for non-protein coding sequence.

To examine the genotypic diversity both within and between accessions, the data from (2) and (3) described above were again converted to single letter codes and an NJ tree constructed as described. For the four selected half-sib families, the male parent of each of the 10 half-sib individuals was identified by testing whether the combination of the female genotype and the candidate male genotype was compatible with the genotype of each progeny HS individual for every marker. For the deduced male parent, SNP markers were deemed to give illegitimate genotypes if the HS genotype could not be derived from the two parent genotypes.

### Population structure

Estimates of the underlying population sub-structure were calculated for the 765 marker loci across the 94 representative mother plants from each of the founder accessions using STRUCTURE v2.3.4 (Pritchard et al. 2000). The analyses were performed using a burn-in period of 500,000 Markov Chain Monte Carlo iterations, with a 500,000 run-length using an admixture model and correlated allele frequencies for *K* sub-populations between 1–6, and 4 independent replications. The output was summarised using the Python script structureharvester.py v0.6.92 (Earl and von Holdt 2012), and the most probable underlying value for *K* was estimated from the ΔK values using the Evanno method (Evanno et al. 2005). The.*indfile for *K* = 4 was used as input for CLUMPP_OSX.1.1.2 (Jakobsson and Rosenberg 2007) using the Fullsearch algorithm, with weighted H and the G similarity statistic. Summarised cluster membership matrices (q values) were generated for both individuals and populations. The KASP SNP data were also analysed using a principal component analysis (PCA). Eigen values were estimated using the singular value decomposition method implemented in the R package (R Core Team 2011) FactoMineR v1.31.4 (Lê et al. 2008). Individuals within the PCA plots were identified based on the proportion of *q*-value cluster membership in the STRUCTURE analyses, where membership is assigned when *q* ≤ 0.25.

### Screening the onion diversity set for FBR resistance

One HS family from each of 83 onion diversity set accessions (Table 1) was screened for FBR resistance using a seedling assay (Taylor et al. 2013). Briefly, this involved soaking onion seeds in a spore suspension (1 × 10^6^ spores mL^−1^) produced from the highly aggressive FOC isolate FUS2 (Lincolnshire, UK) and sowing in modular trays. The highly susceptible cultivar Hystar F_1_ (HY), as well as the partially resistant Ailsa Craig Prizewinner (ACP), was included in the test (Taylor et al. 2013). Non-inoculated controls (seed soaked in water only) were included for each HS. In total, there were four independent replicates of 28 seeds per HS and trays were positioned in a randomised block design in a glasshouse maintained at 25 °C day and 18 °C night with a 16 h photoperiod. Percentage onion seedling survival four weeks post-inoculation was calculated relative to the non-inoculated control treatment for each HS. Significant differences in seedling survival between the HS families were determined using a residual (or restricted) maximum likelihood (REML) analysis following angular transformation of the data (Welham and Thompson 1997) using GENSTAT v12 (VSN International).

A subset of resistant and susceptible HS families was further tested using a mature onion plant assay (Taylor et al. 2013). Briefly, four-week-old onion seedlings were transplanted into 7-cm square pots containing compost infested with FOC isolate FUS2 (1 × 10^5^ cfu g^−1^). Control seedlings were transplanted into compost only. Pots were arranged in a randomised block design in a glasshouse (25 °C day, 18 °C night) with three replicate blocks of 20 plants per HS family. Plants were grown to the mature bulb stage (nine weeks after transplanting), left for two weeks without watering, then weighed and scored for severity of FBR symptoms (Taylor et al. 2013). As the HS families had different daylength responses (Brewster 2008; Taylor et al. 2010), two independent assays were carried out. Firstly, a short-day (12 h photoperiod) assay was set up using two resistant (HO, PO) and two susceptible (EX, NU) HS families along with a partially resistant cultivar (cv. Serrana, Saxena and Cramer 2009). Secondly, a long-day (16 h photoperiod) assay was set up using nine resistant (HYS, CA, DM, GR, ACP, MA, SY, PL, JC) and three susceptible (GRE, HY, ST) HS families. Significant differences between symptom scores were determined using REML and results of seedling and mature plant assays compared using Pearson correlation coefficients (Genstat).

### Screening the onion diversity set for improved seedling vigour

Seedling vigour for 89 HS families was assessed using two different assays, following published protocols with some modifications. The first assay assessed onion germination and initial seedling growth (Finch-Savage et al. 2010). Seed was surface sterilised by immersing in sodium dichlorocyanuric acid (6.2 g, Sigma, UK) in distilled water (100 ml) with two drops of Nonidet P-40 (Sigma, UK) for 10 min on an orbital shaker (200 rpm) followed by three rinses in sterile distilled water. Seeds were then placed on moist sloping filter papers (190 mm wide, Gray and Steckel, 1983) in trays of water and supported at an angle of 30° to the vertical and extending to 100 mm above the water level. Ten seeds from each HS family were placed 55 mm above the surface of the water and slopes arranged in a randomised block design at 15 °C in the dark and observed twice daily for 21 days. Germination and the time for roots to reach 3 cm and shoots to reach 2 cm were recorded. Four replicate experiments were carried out, each with 10 seeds per HS family. The time taken for 50% of viable seeds to complete germination (T50) was calculated. Time to germination for each seedling was subtracted from time for roots to reach 3 cm and shoots to reach 2 cm so that growth times were not confounded by differences in germination times. All data were analysed using REML (Genstat).

The second assay assessed upward growth in strong soil (Finch-Savage et al. 2010). Soil (sandy loam) was dried (80 °C for 3 days) and sieved (2 mm mesh) before adding 60 g of distilled water per kg of soil. Soil was mixed, sealed in bags incubated at room temperature for 2 days. Onion seeds were sterilised as described and then placed on damp chromatography paper in clear plastic germination boxes. Boxes were incubated at 15 °C until a 2 mm radicle was visible (3–8 days depending on the HS). Three layers of soil were placed in 8 × 8 cm pots (250 ml, 150 ml, 150 ml) with each layer compacted using a 7.3 kg weight before adding the next in order to achieve a uniform bulk density. Pre-germinated onion seeds from each HS family (10 per pot) were then placed on the soil surface after which a final layer of soil (150 ml) was added and either compacted or left uncompacted. Pots were sealed in bags in the dark and placed in a randomised block design at 15 °C. Seedling emergence was recorded at 7, 11, 15, 20 and 25 days, and the experiment replicated three times. Angular transformed data for percentage seedling emergence (relative to treatments with a non-compacted top layer) were analysed using REML. The weight of 100 seeds from each HS family was also recorded. Pearson correlation coefficients (Genstat) were calculated to assess whether any of the seedling vigour traits were correlated.

### Association analysis

A preliminary association analysis was carried out to explore if the phenotypic variation for FOC resistance and seedling vigour could be associated with any of the 765 KASP™ markers. Marker data were converted to *A* (*X*), *B* (*Y*) or *H* (heterozygous) and markers sorted into linkage map order (Duangjit et al. 2013) with unmapped markers assigned to a pseudo-group. Kruskal–Wallis rank sum tests were carried out using the mean phenotype data to produce the test statistic (*K*) with an associated significance value using MapQTL® version 6 (Van Ooijen 2009). Markers with a *P* value ≤ 0.005 were considered as being putatively associated with the trait of interest.

## Results

### Development of an onion diversity set

An onion diversity set, consisting of up to 10 half-sib families derived from each of 95 founder genebank accessions, was successfully produced (Table 1), and samples can be sourced from the University of Warwick (https://warwick.ac.uk/fac/sci/lifesci/research/vegin/onion/diversity/).

### SNP genotyping and analysis

Of the 892 SNPs (Table S1) used to genotype mother plants of the HS families from 91 accessions and HY, SE and ACP, 33 were monomorphic, 53 had > 5 missing data points, and 15 had a minor allele frequency < 5%. A further 26 markers had more than one illegitimate genotype in the kinship test described below. The remaining 765 polymorphic markers were used for subsequent genetic analysis.

**Fig. 1:**
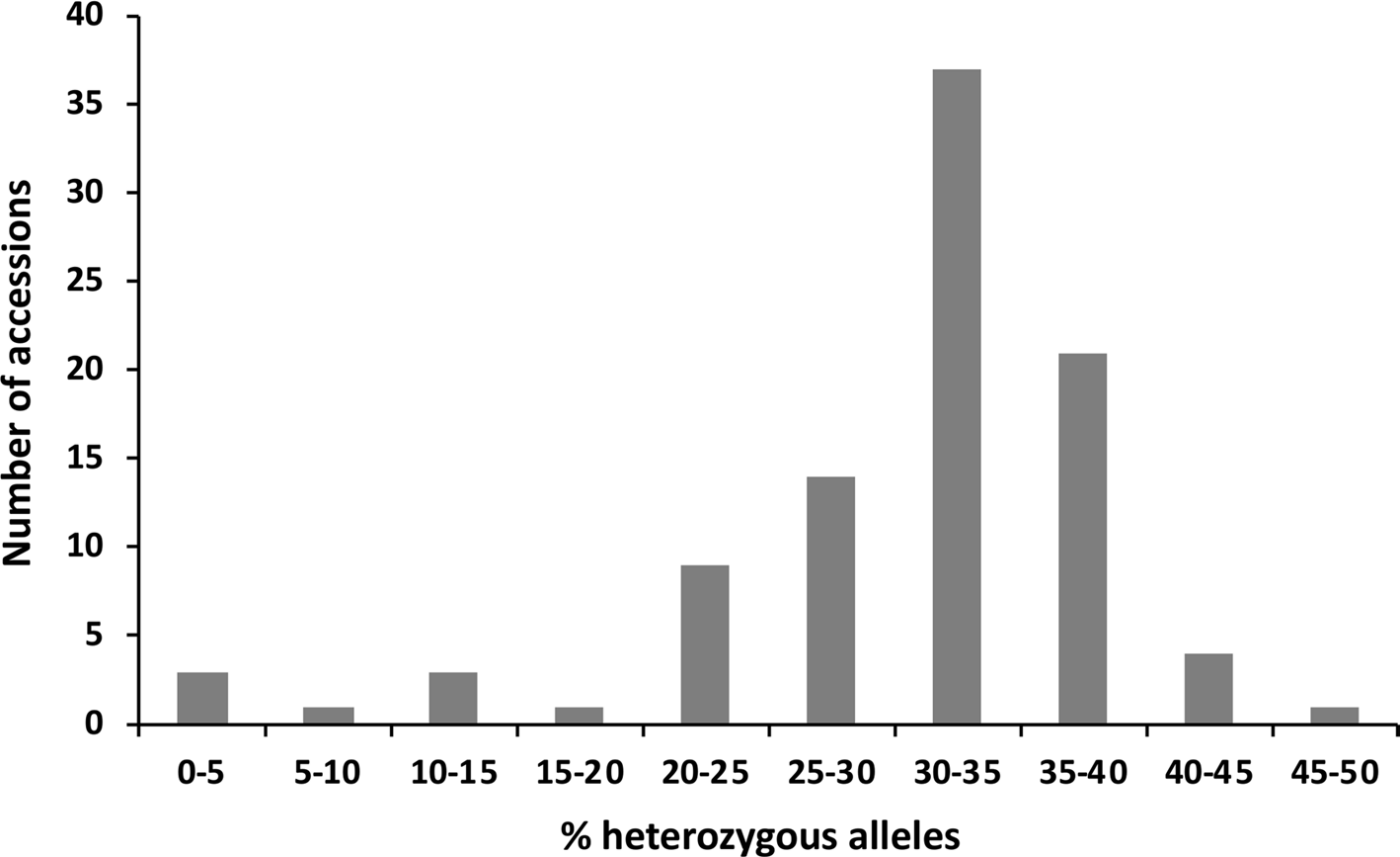
Frequency distribution of recorded heterozygous alleles for 94 accessions in the onion diversity set. A total of 765 KASP markers with minor allele frequency < 5%, with 5 or fewer missing data points, and with zero or one inconsistent alleles in the half-sib female parent analysis (Table S4) were used to determine the proportion of heterozygous alleles

The majority of the onion accessions were moderately heterozygous, with a modal value of 30–35% (Table 1, Fig. 1), consistent with onion being an outbreeding species. The least heterozygous accessions were *A. roylei* (WSR) and *A. fistulosum* (HLW) with 0.31% and 0.55% heterozygosity, respectively. However, these had 128 and 215 missing SNPs, respectively, suggesting that the KASP assays did not detect these loci. Three other accessions had high numbers of missing SNPs, GU (*A. cepa* × *A. fistulosum*), HY and SE with 122, 205, and 219, respectively. By comparison, the mean number of missing SNPs for the remaining onion accessions was only 11. The HS family for GU (*A. cepa* × *A. fistulosum*) was moderately heterozygous (33.3%) likely due to SNPs from homeologs in the amphidiploid, while the most heterozygous *A. cepa* HS was WE (41.6%). As expected, the commercial hybrid cultivar Hystar (HY) was highly heterozygous (47.3%). The least heterozygous *A. cepa* HS was BE (4.9%). Overall, F_1_ hybrid accessions ranged in heterozygosity from 30–40% and were no more heterozygous than many of the open pollinated lines (5–42%, mean 31%). Heterozygous alleles were not restricted to certain SNP markers or regions of the genome, as all but one marker detected heterozygous alleles and 310 markers possessed 30–40% heterozygous alleles. Percentage heterozygosity was not correlated with any of the phenotyping traits (data not shown) and accessions with low heterozygosity still resulted in HS families with good seedling vigour. For instance, accession DM (5.8% heterozygosity) performed well for all vigour measurements.

**Fig. 2:**
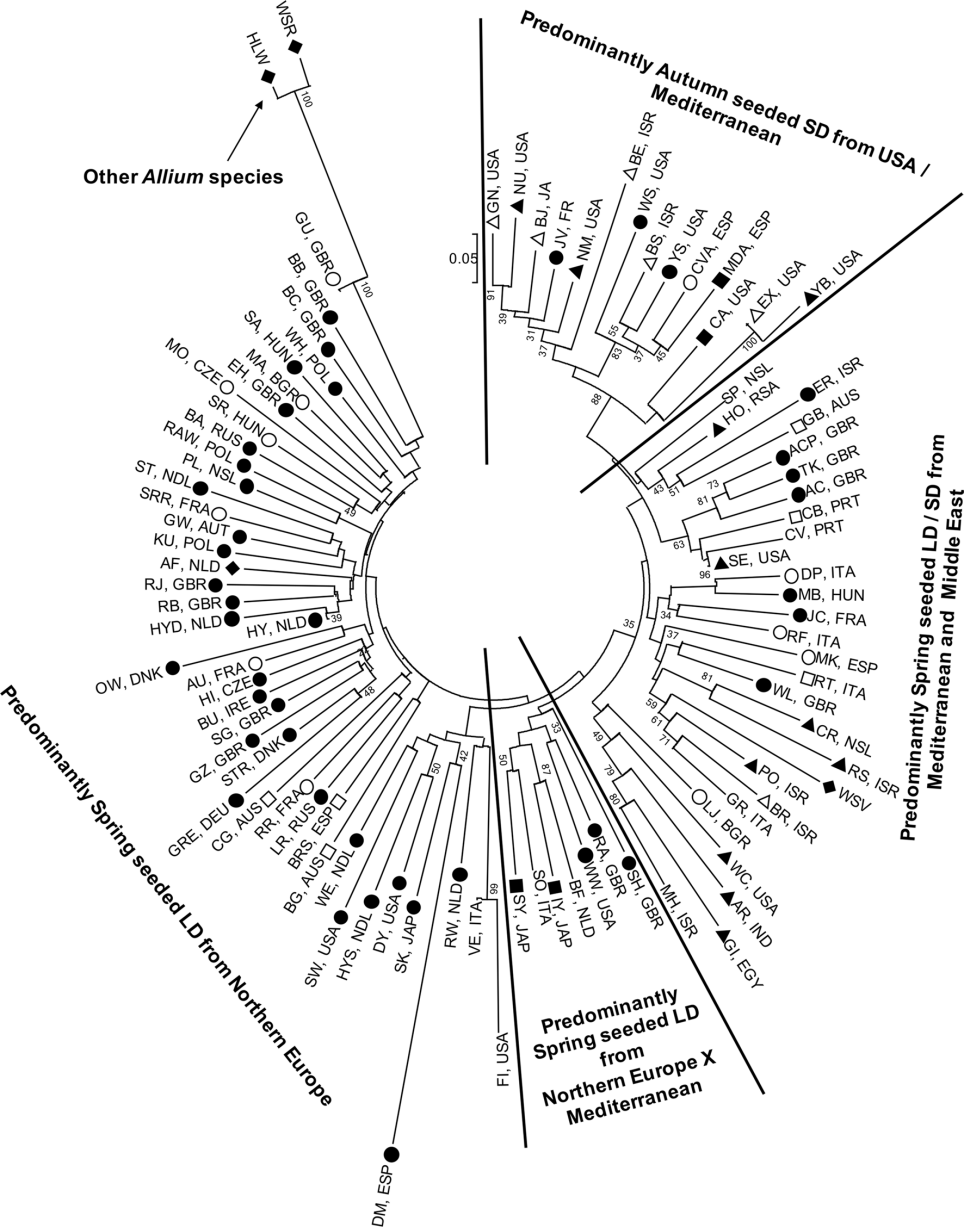
Neighbour-joining phylogenetic tree showing the relationship between 94 accessions from the onion diversity set. 765 KASP™ markers with minor allele frequency < 5%, with 5 or fewer missing data points, and with zero or one inconsistent alleles in kinship analysis (Tables S2 & S4) were used to calculate the tree. Accessions marked by diamonds are wild species. Accessions marked by circles are known to have a long-day requirement for bulbing, those marked with a triangle have a short-day requirement and those with a square have an intermediate day length requirement. Filled symbols indicate that the daylength requirement has been clearly identified for that accession. Bootstrap values are from 1000 replicates and only those > 30% are displayed. Accession names are indicated by the 2- and 3-letter codes as listed in Table 1

The NJ tree for genotypes for the single mother plants from each of the 94 onion accessions showed long branch lengths, indicating a high degree of genetic divergence between accessions (Fig. 2). The wild species *A. roylei* and the bunching onion *A. fistulosum* formed an outgroup which also contained the *A. cepa* × *A. fistulosum* amphidiploid (GU). As expected, shallot was grouped with other onion accessions (Bark and Havey 1995). The wild species *A. vavilovii* grouped with *A. cepa* lines, suggesting that this accession may be mislabeled or is a feral *A. cepa*. The *A. cepa* accessions did not group by country of origin, most likely because SD, ID and LD cultivars can be grown in the same region depending on autumn or spring plantings. However, there was some grouping according to daylength requirements and production area. The largest grouping was predominantly composed of accessions that are spring-seeded in LD regions such as Northern Europe, Japan, and North America while another group generally comprised autumn-seeded SD cultivars grown in the Mediterranean, Middle East and India which were subsequently introduced to the southern USA (Fig. 2). A third group predominantly comprised both LD and SD spring-seeded onions from the Mediterranean and the Middle East.

**Fig. 3:**
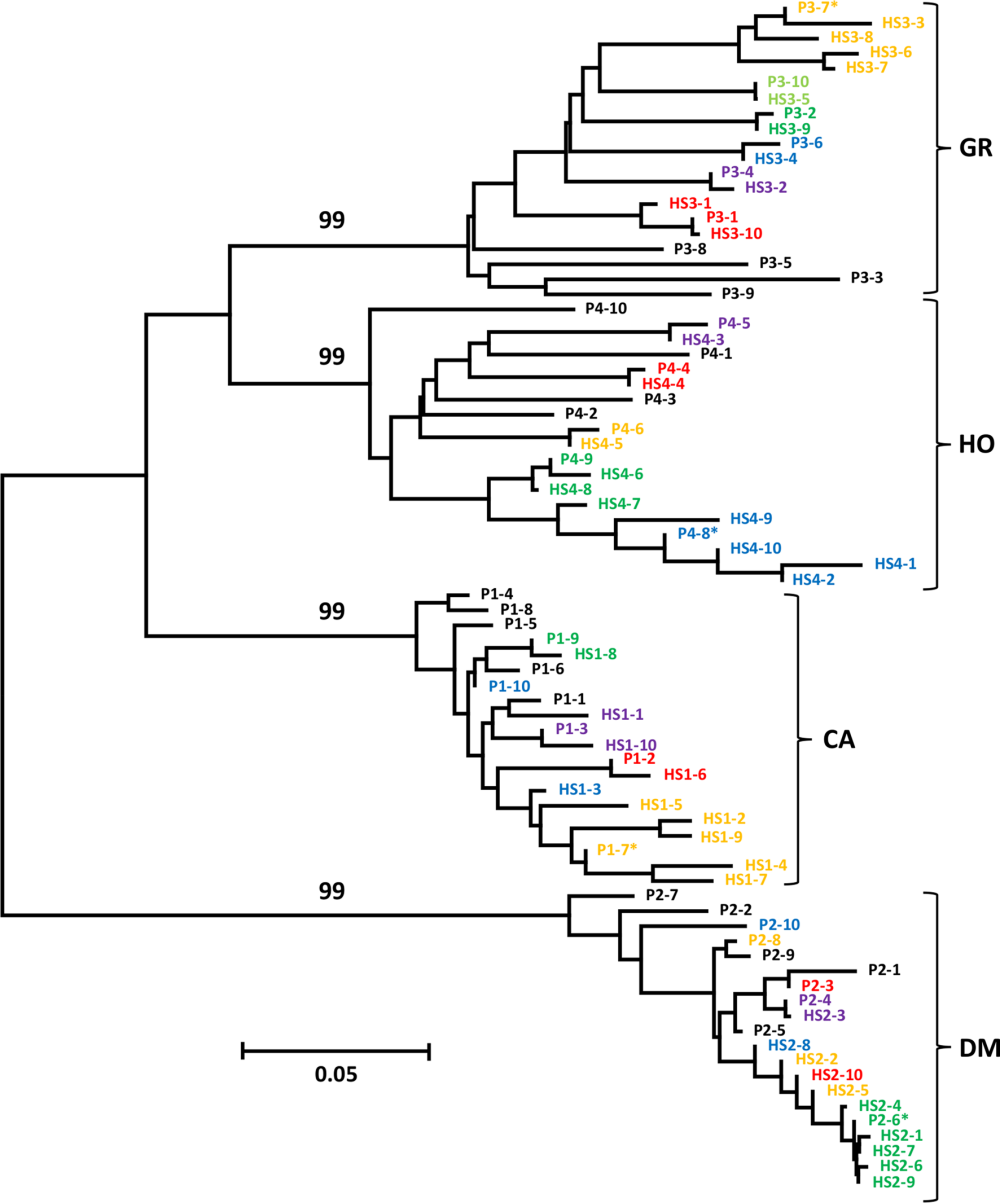
Neighbour-joining tree of half-sib parent lines and half-sib individuals for four onion accessions. Up to ten half-sib parent lines and half-sib individuals were genotyped and a subset of 568 of the 765 KASP™ markers used to calculate the tree. P1_1 indicates parent 1 of HS family 1 while HS1_1 indicates half-sib individual 1 of accession 1 etc. The percentage bootstrap values for 1000 bootstrap repetitions are indicated on the primary branches marking each of the four accessions. HS1 = Candy F1 (CA), HS family 2 = Downy Mildew Resistant Selection (DM), HS family 3 = Giant Rocca Brown (GR) and HS family 4 = Hojem (HO). *Indicates the female parent of the ten HS individuals and colours indicate the deduced male parent (Table S4)

The NJ tree for ten mother plants from each of the four selected onion founder accessions (CA, DM, GR and HO) as well as the ten individuals from a single half-sib family from one of these mother plants indicated extensive diversity both between and within HS families from the same parent accession (Fig. 3, Table S2). This shows that the genetic variability within a single onion accession can be high, owing to its out-crossing nature. However, greater genetic variation was observed between accessions than within an accession and all mother plants and half-sib individuals from the same founder onion accession clustered in the same clade indicating that an onion accession is still a recognisably discrete genetic entity (Fig. 3). Comparable levels of heterozygosity for each individual plant within a HS family were also observed (Table S3). Based on the lowest number of conflicting alleles, it was possible to deduce the male parent for each of the individuals genotyped from a single HS family (Table S4). It was found that 40–50% of the half-sib individuals were the result of self-pollination of the mother plant (5/10 for CA, 5/10 for DM, 4/10 for GR and 4/10 for HO). In addition, HS individuals were seen to cluster with their deduced male parent in the phylogenetic tree (Fig. 3).

### Population structure

**Fig. 4:**
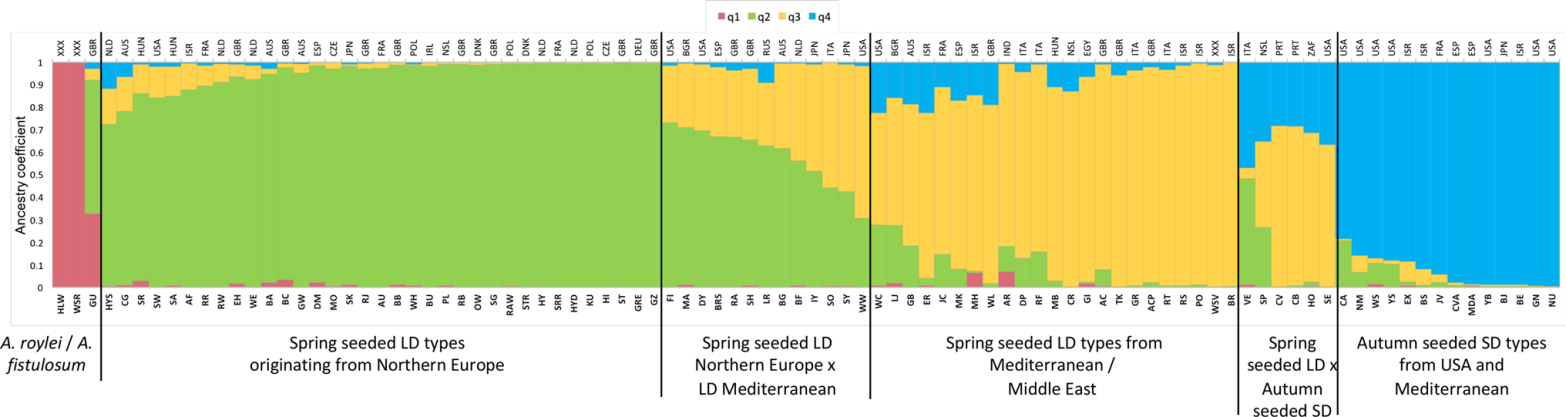
Population structure for 94 accessions from the onion diversity set following analysis of KASP™ SNP data using STRUCTURE. Data plot is of estimated sub-population membership when *K* = 4. Data are sorted by *q* value membership (*q*1 = red, *q*2 = green, *q*3 = yellow, *q*4 = blue). Accessions are grouped by *q* value membership using a minimum threshold of ≥ 0.25. Accession names are indicated by the 2- and 3-letter codes as listed in Table 1; for reference, three letter country codes are presented on the secondary x-axis

**Fig. 5:**
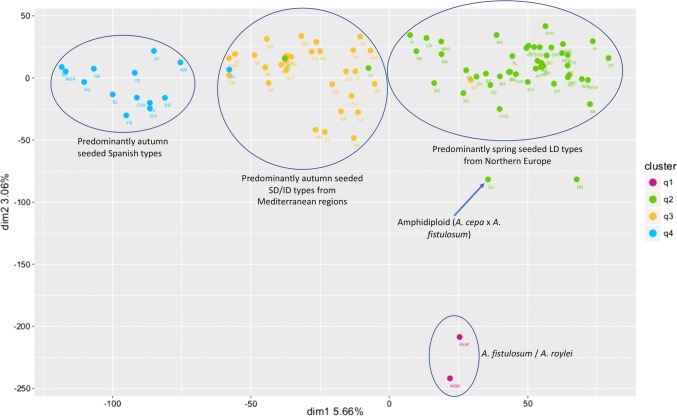
Principal component analysis of KASP™ SNP data from the onion diversity set. Individuals factor map is estimated using HS individuals from 94 accessions genotyped at 765 loci. Individuals are coloured based on their *q* value cluster membership (*q *≥ 0.25) following the STRUCTURE analysis (Fig. 4). Admixed clusters, are ordered in descending cluster membership, i.e. the highest *q* value first. Accession names are indicated by the 2- and 3-letter codes as listed in Table 1. The accession DM (Downy Mildew Resistant Selection) was placed close to the amphidiploid

STRUCTURE analysis indicated that the best estimation of sub-population membership was *K* = 4. Clear clusters were observed, with *q*2, *q*3, and *q*4 explaining the majority of the variation (Fig. 4), corresponding to the groupings observed in the NJ tree (Fig. 2). Accessions from *A. fistulosum* (HLW) and *A. roylei* (WSR) had *q*1 as the dominant membership, with the *A. cepa* × *A. fistulosum* hybrid GU also having > 25% *q*1 membership, reflecting its pedigree. Cluster *q*2 contained spring-seeded LD accessions originating from northern Europe and introduced into LD production regions such as New Zealand (PL) or USA (SW). Cluster *q*3 contained LD, SD and ID accessions that are generally autumn-seeded and grown in the Mediterranean region. Cluster *q*4 was dominated by accessions from the USA, many of which are autumn-seeded SD onions (e.g. NU, GN, BE, BJ, and YB) originating from Spanish populations such as Babosa or Valenciana (Havey and Ghavami 2018). The broad groupings identified from the STRUCTURE analysis were also present in the complementary principal component analysis of the SNP data (Fig. 5). Individuals that have dominant *q*2, *q*3, or *q*4 cluster membership were clearly separated in dimension 1, with varying combinations of cluster memberships interspersed between these groupings. The two *A. fistulosum* (HLW) and *A. roylei* (WSR) accessions in cluster *q*1 were clearly resolved in dimension 2, once again highlighting how different these are from the rest of the diversity set. Interestingly, Candy F_1_ (CA) is derived from an LD × SD cross and was placed between q3 and q4 on the PCA plot.

### Screening the onion diversity set for FBR resistance

**Fig. 6:**
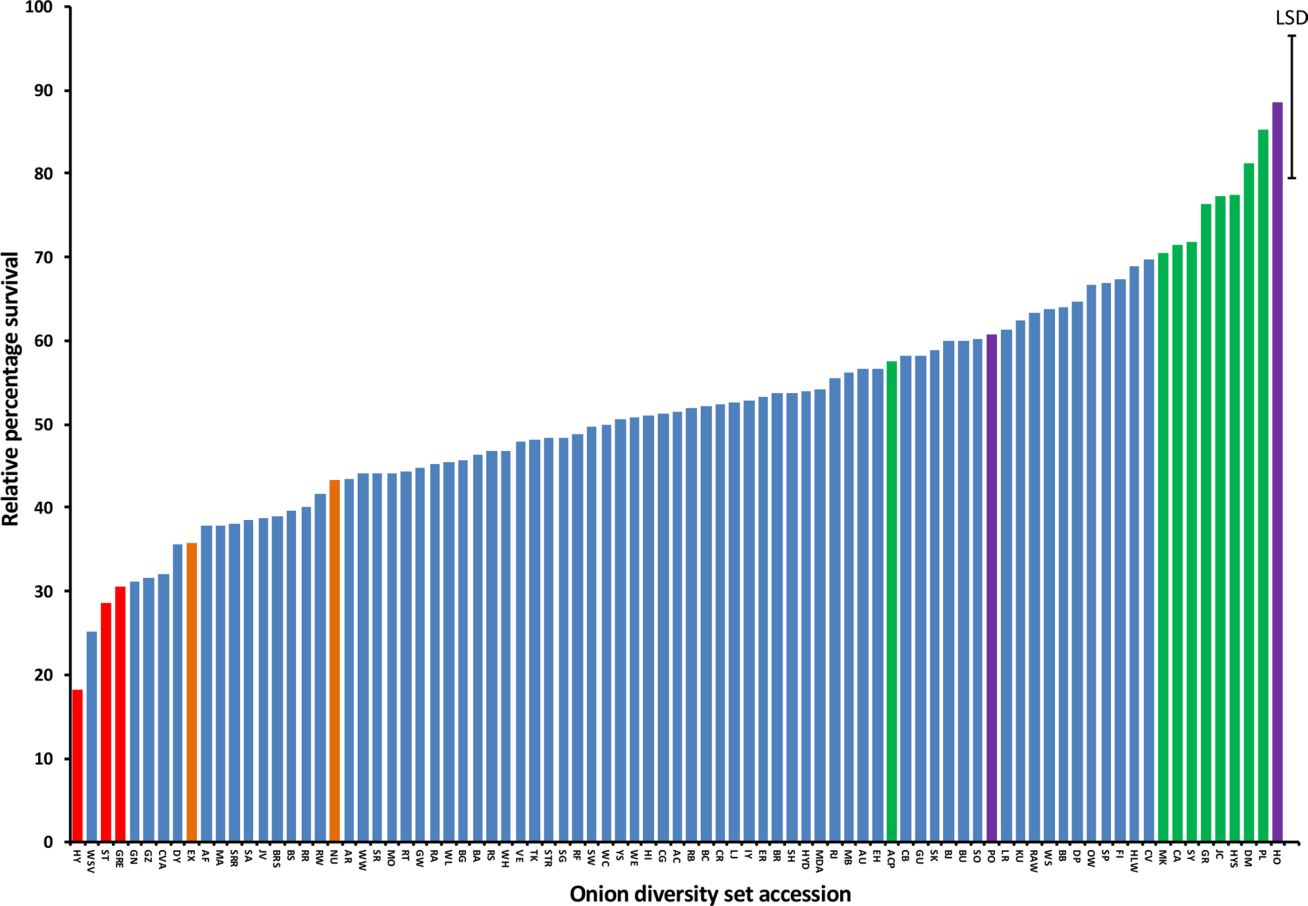
Percentage seedling survival (relative to the untreated control, angular transformed data) for 85 onion diversity set accessions following screening for resistance to *Fusarium oxysporum* f.sp *cepae* (isolate FUS2). Error bar indicates maximum LSD (5% level). Green bars, resistant LD/ID accessions used in the mature plant assay; purple bars, resistant SD accessions used in the mature plant assay; red bars, susceptible LD/ID accessions used in mature plant assay; orange bars, susceptible SD accessions used in mature plant assay. Accession names are indicated by the 2- and 3-letter codes as listed in Table 1

In the seedling assay, significant variation (*P* < 0.001) in relative plant survival compared to the uninoculated controls (Fig. 6), ranging from 25.2% (WSV) to 88.6% (HO), was observed. Six accessions (HO, PL, DM, HYS, JC and GR) showed a significantly greater level of FOC resistance (76.4–88.6%) than the partially resistant ACP (57.6%). HS families from 78 accessions were significantly more resistant than the susceptible control cultivar Hystar (18.2%).

**Fig. 7:**
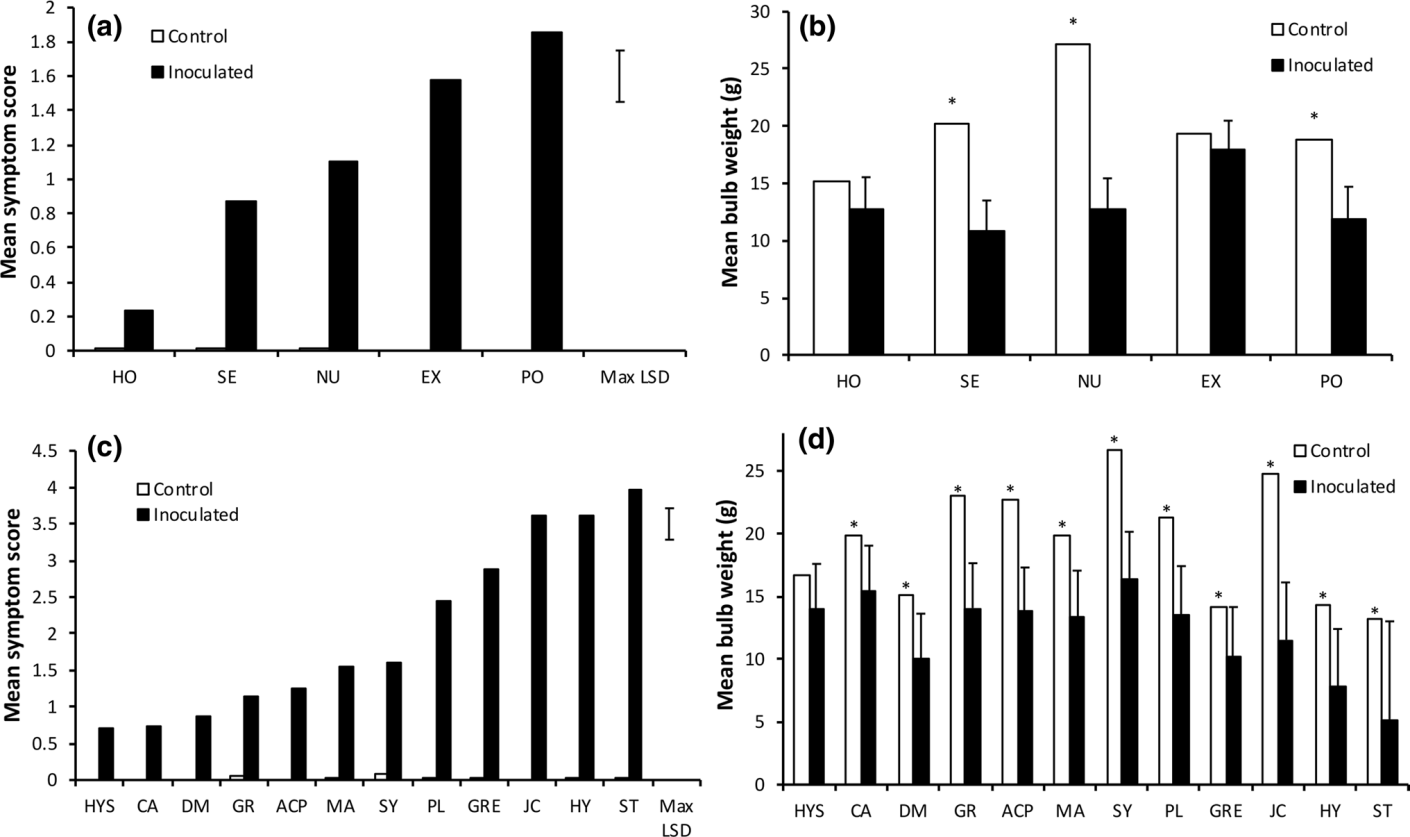
Severity of Fusarium basal rot following screening of 17 accessions from the onion diversity set for resistance to *Fusarium oxysporum* f.sp *cepae* (isolate FUS2) using a mature plant assay; **a** mean symptom score for SD assay; **b** mean bulb fresh weight for SD assay; **c** mean symptom score for LD assay. **d** Mean bulb fresh weight for LD assay. Error bars indicate LSDs (5% level) for comparing lines (**a**, **c**) or comparing inoculated and control (**b**, **d**). *Indicates a significant difference compared to the control. Accession names are indicated by the 2- and 3-letter codes as listed in Table 1

FBR resistance was confirmed in the mature plant assays. Within the SD material, HO which was the most resistant accession in the seedling assay had a significantly lower FBR (mean score 0.23) than the other three accessions tested (NU, EX, PO; Fig. 7a; *P* < 0.001) as well as the cv. Serrana (mean score 0.87). The most susceptible accession was PO (mean score 1.85) which was partially resistant in the seedling assay. Significant reductions in bulb weight compared to the uninoculated control (*P* < 0.001) were observed for Serrana, NU and PO (Fig. 7b) but not for HO. Although no significant reduction in bulb weight was observed for EX, this data do not include five plants which died due to FOC infection.

In the LD assay, HS families from seven (HYS, CA, DM, GR, ACP, MA and SY) of the nine onion accessions selected as being resistant in the seedling assay had significantly (*P* < 0.001) lower mean FBR disease scores (0.7–1.6) than the susceptible HY control (mean disease score 3.6, Fig. 7c). The remaining two accessions, PL and JC, had mean disease scores of 2.4 and 3.6, respectively. The highest level of FBR resistance was shown by HYS, CA and DM (mean disease scores of 0.72, 0.75 and 0.88, respectively), and for HYS there was also no significant reduction in bulb weight (Fig. 7d). HS families from the two accessions selected as being susceptible in the seedling assay (ST, GRE) were also susceptible in the mature plant assay with mean disease scores of 4.0 and 2.9, respectively. Results across all LD and SD data indicated that the seedling test correlated well with the mature plant test with a strong negative correlation (*n* = 16, *r* = − 0.57, *P* = 0.020) between percentage seedling survival in the seedling assay and disease score in the mature plant assay (Fig. S1). HS families from the seven accessions with the highest level of FOC resistance were in different clades of the phylogenetic tree (Fig. 2), suggesting multiple independent loci linked to a resistance phenotype.

### Screening the onion diversity set for improved seedling vigour

**Fig. 8:**
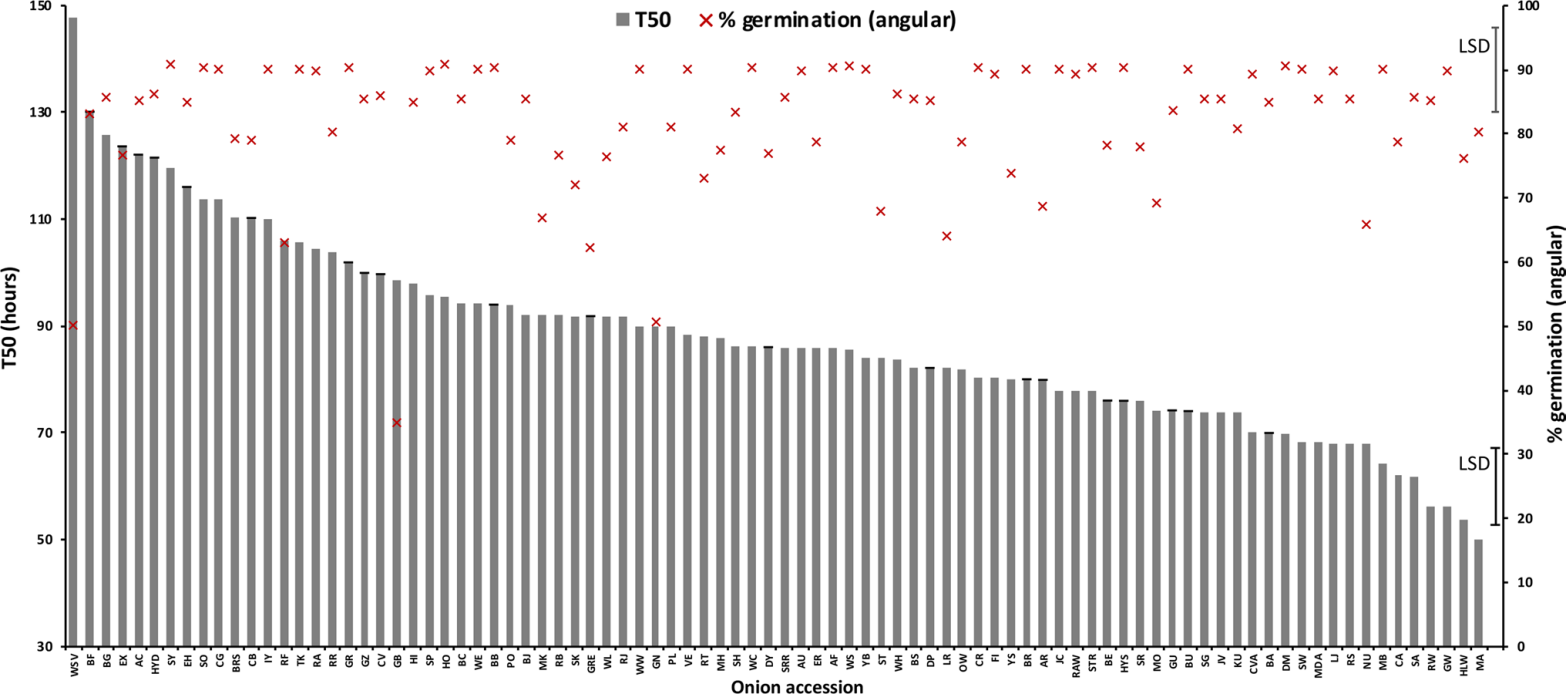
Percentage germination (angular transformed) and time to 50% viability (T50) for 87 accessions from the onion diversity set. Error bars indicate LSD (5% level). Accession names are indicated by the 2- and 3-letter codes as listed in Table 1

**Fig. 9:**
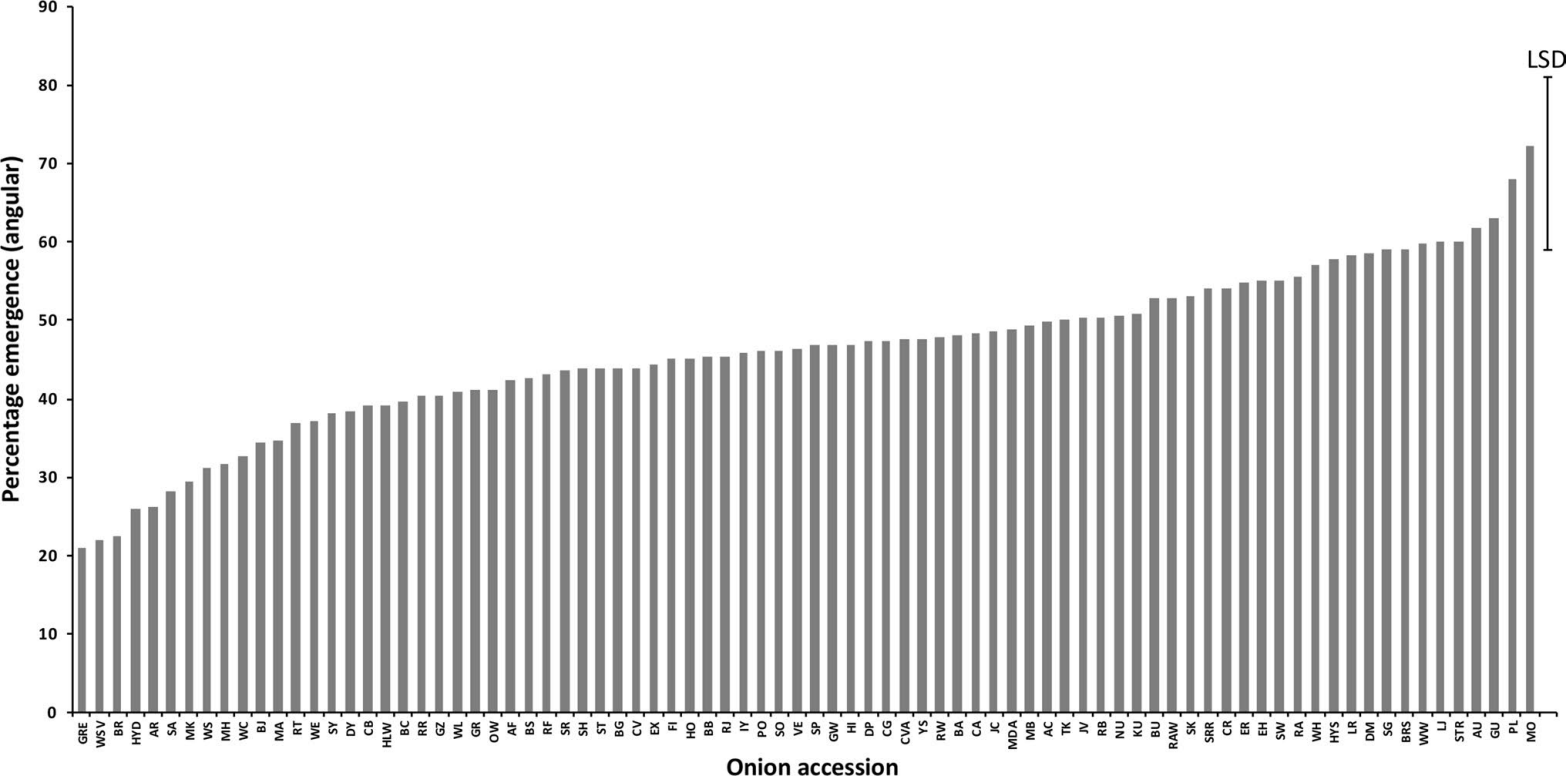
Percentage emergence in compact soil (angular transformed data, relative to a non-compacted top layer control) for 82 accessions from the onion diversity set. Error bar indicates LSD (5% level). Accession names are indicated by the 2- and 3-letter codes as listed in Table 1

Significant differences were observed between 83 HS families tested for root growth rate, shoot growth rate, T50, percentage germination and emergence from compacted soil (*P* < 0.001 for all traits). T50 values ranged from 50 h (MA) to 148 h (WSV, Fig. 8). Percentage germination was generally high across accessions indicating that seeds were of high quality, but this was not correlated with T50 (*n* = 87, *r* = − 0.12, *P* = 0.25, Table 2) showing that the two traits are independent. The time for roots to reach 3 cm ranged from 94 h (HYS) to 195 h (WSV, Fig. S2), while the time taken for shoots to reach 2 cm ranged from 179 h (KU) to 279 h (WSV, Fig. S2). HS families from accessions HYS, DP and WE showed very high levels of vigour for both root and shoot growth rate. There was a strong positive correlation between root and shoot growth (*n* = 87, *r* = 0.59, *P* < 0.0001; Table 2). A wide range of responses were also observed for seedling emergence in compacted soil across accessions, varying from 20.9% (GRE) to 72.3% (MA) relative to the uncompacted control (Fig. 9). This trait was strongly positively correlated with seed weight (*n* = 82, *r* = 0.46, *P* < 0.0001) and moderately positively correlated with percentage germination (*n* = 82, *r* = 0.36, *P* < 0.001).

**Table 2:** Pearson correlations coefficients (*r*) between all the measured traits in the onion diversity set

	Root time	Shoot time	Fusarium resistance	Seed weight	T50	% Germination	% Emergence in compact soil
Root time		0.59***	− 0.37**	− 0.19	0.16	− 0.36**	− 0.35*
Shoot time	0.59***		− 0.19	− 0.26	0.15	− 0.25	− 0.27
Fusarium resistance	− 0.37**	− 0.19		0.26	− 0.06	0.36**	0.18
Seed weight	− 0.19	− 0.26	0.26		− 0.01	0.36**	0.46***
T50	0.16	0.15	− 0.06	− 0.01		− 0.12	− 0.22
% Germination	− 0.36**	− 0.25	0.36**	0.36**	− 0.12		0.23
% Emergence in compact soil	− 0.35*	− 0.27	0.18	0.46***	− 0.22	0.23	

Root time = time for root to reach 3 cm; shoot time = time for shoot to reach 2 cm; Fusarium resistance = percentage survival in seedling assay; T50 = time to reach 50% germination

^*^P < 0.005; **P < 0.001; ***P < 0.0001

Correlation coefficients were also calculated for all other combinations of traits measured across the accessions in the diversity set (Table 2). A moderate correlation between survival in the FBR seedling assay and time for root to reach 3 cm was observed (*n* = 83, *r* = − 0.37, *P* < 0.001), suggesting a possible general trend between fast root growth and FOC resistance. There was also a weak correlation between FOC resistance and percentage seed germination. However, the accession LJ performed extremely well for all vigour parameters tested yet was susceptible to FOC, suggesting that FBR resistance is a discrete independent trait and not a pleiotropic effect of increased seedling vigour.

### Preliminary association analysis

The Kruskal–Wallis tests identified SNP markers that were significantly associated with each of the tested traits (Table 3). For FOC resistance, five markers were identified, three of which are mapped (Duangjit et al. 2013), indicating possible quantitative trait loci (QTL) on chromosomes 1, 6B and 8. A large number of SNP markers were found to be associated with root or shoot growth with two unmapped markers associated with both. Two SNP markers (both unmapped) were significantly associated with growth in compact soil and were also associated with shoot growth. Since this method is a single marker test and does not use genomic control, a stringent *P *value of ≤ 0.005 was used to minimise the risk of false positives.

**Table 3:** SNP markers showing a significant association with traits evaluated in the onion diversity set. KW score refers to the Kruskal–Wallis score with higher numbers indicating a stronger association. Matching numbers indicate markers linked to multiple traits

Trait	Linked markers	Map position^a^	KW score	*P* value
Fusarium resistance	^1^I30594_1021	Ch8, 1.1	13.18	0.005
	i34519_442	Ch6B, 30.2	12.99	0.005
	^2^c00676_1004	1, 149.8	12.60	0.005
	i33746_1093	Unmapped	10.93	0.005
	i33439_640	Unmapped	10.73	0.005
Germination %	I16136_1083	Unmapped	13.10	0.005
	i28645_2547	Unmapped	11.61	0.005
	i37348_328	Unmapped	16.16	0.0005
	i34666_385	Unmapped	11.17	0.005
	i29175_343	Ch4B, 64.6	10.71	0.005
	2c00676_1004	Ch1, 149.76	15.22	0.0005
	4i31728_1335	Unmapped	14.32	0.001
	1i30594_1021	Ch8, 1.11	14.00	0.001
	i32829_1057	Ch4B, 68.49	13.36	0.005
	i29044_2564	Unmapped	11.33	0.005
	i40786_596	Ch7, 66.17	11.31	0.005
	i30880_1388	Ch6B, 9.31	11.16	0.005
	i28964_1550	Unmapped	18.35	0.0005
	i26540_301	Ch4B, 145.53	16.21	0.0005
	^3^i26524_1202	Unmapped	15.38	0.0005
	i19082_1721	Unmapped	14.85	0.001
	i22582_277	Unmapped	14.80	0.001
	i39121_540	Unmapped	13.69	0.005
	i31050_188	Unmapped	13.41	0.005
	i32924_1099	Unmapped	13.21	0.005
	^4^i31728_1335	Unmapped	12.67	0.005
	i35169_768	Unmapped	12.21	0.005
	c00172_269	Unmapped	12.00	0.005
	i39194_488	Ch1, 0.0	11.99	0.005
	c00456_208	Unmapped	11.93	0.005
	i32340_410	Unmapped	11.82	0.005
	^5^i30323_747	Unmapped	11.80	0.005
	i30732_1102	Unmapped	11.72	0.005
	i29462_1710	Unmapped	11.63	0.005
	i36037_559	Unmapped	11.14	0.005
	i33279_237	Ch2, 59.36	11.01	0.005
	^6^i31632_1362	Unmapped	10.68	0.005
Emergence compact %	^6^i31632_1362	Unmapped	18.81	0.0001
	5i30323_747	Unmapped	10.74	0.005

^a^Map positions (Chromosome [Chr]/cM) according to Duangjit et al. (2013)

## Discussion

Conserving biodiversity is critical for the future sustainability of crop production by providing diverse alleles for exploitation in breeding programmes (Keller et al. 2013). Central Asia is the centre of diversity for many *Alliums* and has been a source of wild species exhibiting potentially useful traits (Simon 2005; Pandey et al. 2008; Baldwin et al. 2012a; Havey and Ghavami 2018). However, the difficulty in introgressing traits means that it can take as long as 20 years to breed these traits into a commercial cultivar (Scholten et al. 2007; Chuda and Adamus 2008). For this reason, the diversity set reported here was predominantly created using *A. cepa* accessions. Many collections contain large numbers of *Allium* accessions (Khosa et al. 2016), too many to feasibly phenotype for traits of interest. To address this, diversity core collections are often developed which aim to represent the diversity in a crop in a manageable number of accessions (Walley and Moore 2015). A recent study examined genetic diversity in a set of 73 onion accessions consisting of commercial cultivars (3 countries) and Italian landraces (Vilano et al. 2019). Crops such as barley and lettuce are naturally inbreeding and as such diversity sets derived from genebank accessions are more amenable to association mapping (Houston et al. 2014; Walley et al. 2017). In other crops, such as vegetable brassicas, value has been added to diversity sets by generating homozygous lines (Walley et al. 2012; Hatzig et al. 2015; Havlickova et al. 2018).

We developed a unique onion diversity set, designed to capture diversity of this crucially important vegetable crop, comprising 90 *A*. *cepa* accessions from 23 different countries, together with four close relatives and an *A. cepa* × *A. fistulosum* hybrid. As onion exhibits severe inbreeding depression (Brewster 2008), the generation of a homozygous diversity set was not possible; therefore, value was added by creating seed stocks of multiple HS families for each accession. Seed is stored under Genebank conditions meaning it will remain viable for decades (FAO 2013). Using KASP™ markers (Duangjit et al. 2013), we determined that genetic diversity was captured both across the diversity set and within accessions, thus providing an invaluable resource for future research and breeding. Our analysis revealed that 765 of the published SNPs were informative for this diversity set, whereas a recent publication on 73 onion accessions (Vilano et al. 2019) tested only 400 of these SNPs, finding 375 to be informative. The observation that germination rate was high indicates that vigour was maintained due to the approach of generating HS families and the KASP™ marker data indicated that this is due to heterozygosity being maintained. Despite this, the analysis of individuals within HS families and between mother plants (Fig. 3) showed that the genetics of the original accessions were still maintained as individuals and parents from a HS family clustered together. The KASP™ markers revealed a moderate level of heterozygosity (mode 30–35%) in the HS families with the exception of *A. roylei* (WSR) and *A. fistulosum* (HLW). This is likely due to the fact that the KASP™ markers were designed to SNPs between two *A. cepa* populations (Duangjit et al. 2013) and are not polymorphic in other *Allium* species. A previous study using 1226 SNP markers reported slightly lower heterozygosity (23.5%) in 14 OP onion populations (Havey and Ghavami 2018) while another reported 22% heterozygosity using 166 SSR markers to assess 24 populations (Baldwin et al. 2012a). Conversely, an earlier study using 56 SSR markers showed that the median level of heterozygosity in 72 OP onion populations was 70% (McCallum et al. 2008). A moderate to high level of heterozygosity is consistent with onion being an outbreeding species, and relatively high levels (up to 20%) of heterozygosity have even been observed in inbred onion lines (Bradeen and Havey 1995; Duangjit et al. 2013). The present study is the first to examine heterozygosity across a large panel of diverse onion accessions. We observed a small number of onion accessions which were not highly heterozygous (e.g. BE and DM) which might have been expected to lack vigour due to inbreeding depression (Jones and Davis 1944; Brewster 2008). However, while BE has slow root and shoot growth rates, DM did not suffer any loss of vigour suggesting that either this low level of heterozygosity (5.8%) is sufficient to retain vigour or this accession is heterozygous at the loci needed to maintain vigour.

Genotyping of 91 diversity set accessions showed that they were related to each other predominantly through long phylogenetic branches indicating a low degree of genetic redundancy. There was also considerable variation within the four HS families analysed, indicating that an onion cultivar is a population of non-identical but genetically related individuals. This has implications for introgressing traits into a new cultivar as attention will need to be paid to maximising the frequency of beneficial alleles into as many individuals as possible. The results from the phylogenetic and population structure analyses supported the historic breeding of onion for adaption to daylengths at different latitudes (Brewster 2008; Taylor et al. 2010), highlighting the importance of conserving a range of SD, LD and ID onion lines in Genebank collections. Furthermore, both analyses grouped a shallot line (AF, *A. cepa var. ascalonicum*) with *A. cepa* supporting the previous assertion that this species should be classified as *A. cepa* (Brewster 2008; McCallum et al. 2008).

The onion diversity set was successfully screened for resistance to FBR, a global problem for onion growers (Cramer 2000; Taylor et al. 2016). Previous studies have identified partial resistance or ‘tolerance’ to FBR in *A. cepa,* but high-level resistance is lacking (Saxena and Cramer 2009; Taylor et al. 2013; Gei et al. 2014). While high levels of FBR resistance have been identified in other *Allium* species (Galván et al. 2008; Rout et al. 2016), the problems associated with introgression of this trait into *A. cepa* have prevented this resistance being exploited. We identified high levels of FBR resistance in a number of *A. cepa* accessions from different genetic backgrounds which in the future may be particularly valuable for ‘stacking’ of resistance genes for improved durability. Furthermore, FBR resistance was identified in both SD and LD material thus simplifying breeding for different latitudes. Our diversity set also included an *A. fistulosum* accession (HLW), a species which has previously been shown to have higher resistance to FOC than *A. cepa* (Abawi and Lorbeer 1971a; Holz and Knox-Davies 1974; Galván et al. 2008). However, in the seedling assay, HS families from several *A. cepa* accessions were as resistant as HLW, while HO was significantly more resistant. This confirms the utility of the onion diversity set in identifying high levels of resistance to FBR in *A. cepa*. A significant correlation was observed between seedling and mature plant assays, suggesting that the former can be used as a rapid phenotyping approach to screen large populations for resistance. This confirms our previous work (Taylor et al. 2013) and that of others (Retig et al. 1970; Özer et al. 2004) who confirmed that there is a strong correlation between the results of glasshouse and field resistance screening. However, we suggest that seedling resistance should be confirmed using a mature plant assay as this tests resistance at the bulb stage. While resistance was only confirmed using a single FOC isolate, this isolate was shown to be highly virulent and previous studies suggested that there is no cultivar × isolate interaction (Taylor et al. 2013; Gei et al. 2014). A partial correlation was observed between FBR resistance and root growth rate, suggesting that these two traits may be linked. However, this was not always the case as HS family LJ scored highly in all vigour assays but did not exhibit FOC resistance. In previous research, *Allium* accessions with denser root systems had higher resistance to FOC (Galván et al. 2008). Developing SNP markers linked to FBR resistance in onion will allow rapid breeding using marker-assisted selection, something which would be greatly beneficial due to the biennial life cycle of onion. Association analyses identified three SNPs significantly correlated with FBR resistance. While this result suggests an excellent chance of identifying QTL in the future, linkage of these markers to FBR resistance loci needs to be confirmed using biparental haploid mapping populations derived from resistant × susceptible crosses.

Seedling vigour is a critical trait for sustainable crop production (Finch-Savage et al. 2010; Finch-Savage and Bassel 2016), and this is the first time that a diverse range of onion accessions have been examined for this trait. Highly vigorous accessions were identified and rapid and high-throughput phenotyping assays developed, something which is often the ‘bottleneck’ for genetic analyses. The protocol used here aligns with the ISTA seed testing protocols for onion (ISTA 2013). It was apparent that many of the individual components that constitute ‘vigour’ are correlated, and potentially under common genetic control with the notable exception of T50 which was a distinct trait. This is an important finding as fewer parameters could be selected to assess vigour of different seed lots. Of particular note is the strong correlation between seed weight and emergence in compact soil. A similar observation was made for other crops (Finch-Savage et al. 2010; Finch-Savage and Bassel 2016) meaning that seed weight can be used as a predictor of emergence in compact soil, which is relevant to production in soils liable to capping. All seedling vigour parameters measured here will potentially influence establishment in the field and the HS family LJ performed well across all of them, providing an excellent source of pre-breeding material for this trait.

Kruskal–Wallis tests identified SNP markers that may be linked to both FBR resistance and seedling vigour. In the absence of estimates of linkage disequilibrium, these markers represent preliminary associations. Since this method is a single marker test without a genomic control, a stringent *P *value of ≤ 0.005 was used to reduce false positives. When the genome sequence of onion becomes available, we will be able to align the markers used in this research, and generate additional markers to gain a representative sampling of the genome, and estimate the underlying linkage disequilibrium. Until then, the putative marker-trait associations identified in this work are a useful starting point for future selection in breeding programmes, in combination with the phenotypic variation for FBR resistance and seedling vigour that we have discovered.

## Electronic supplementary material

Below is the link to the electronic supplementary material.

Supplementary file1 (PDF 125 kb)

Supplementary file2 (XLSX 577 kb)

Supplementary file3 (DOCX 168 kb)
