# Supplementary material for: Assembly and characterisation of a unique onion diversity set identifies resistance to Fusarium basal rot and improved seedling vigour
Source: Theor Appl Genet. 2019 Sep 13;132(12):3245–64. doi: 10.1007/s00122-019-03422-0 (PMC6820603; doi:10.1007/s00122-019-03422-0)
Supplement: Supplementary file 1 — Supplementary file1 (PDF 125 kb) [file 122_2019_3422_MOESM1_ESM.pdf]

Assembly and characterisation of a unique onion diversity set identifies resistance to Fusarium basal rot and improved seedling vigour

Theoretical and Applied Genetics

Taylor A*, Teakle GR, Walley PG, Finch-Savage WS, Jackson AC, Jones JE, Hand P, Thomas B, Havey MJ, Pink DAC, Clarkson JP.

**Warwick Crop Centre, School of Life Sciences, University of Warwick, andrew.taylor@warwick.ac.uk*

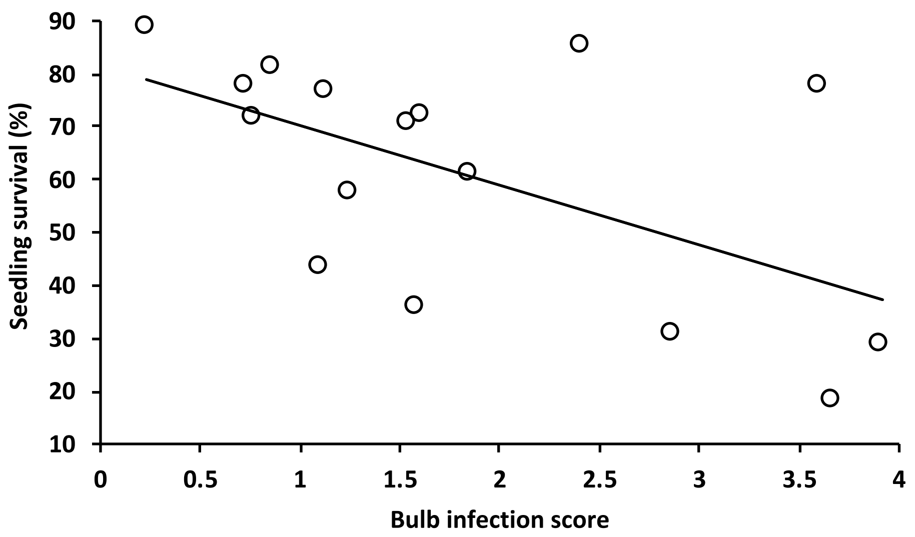

Fig. S1: Correlation between seedling and mature plant assays used to inoculate an onion diversity set with *Fusarium oxysporum* f. sp. *cepae* (isolate FUS2).

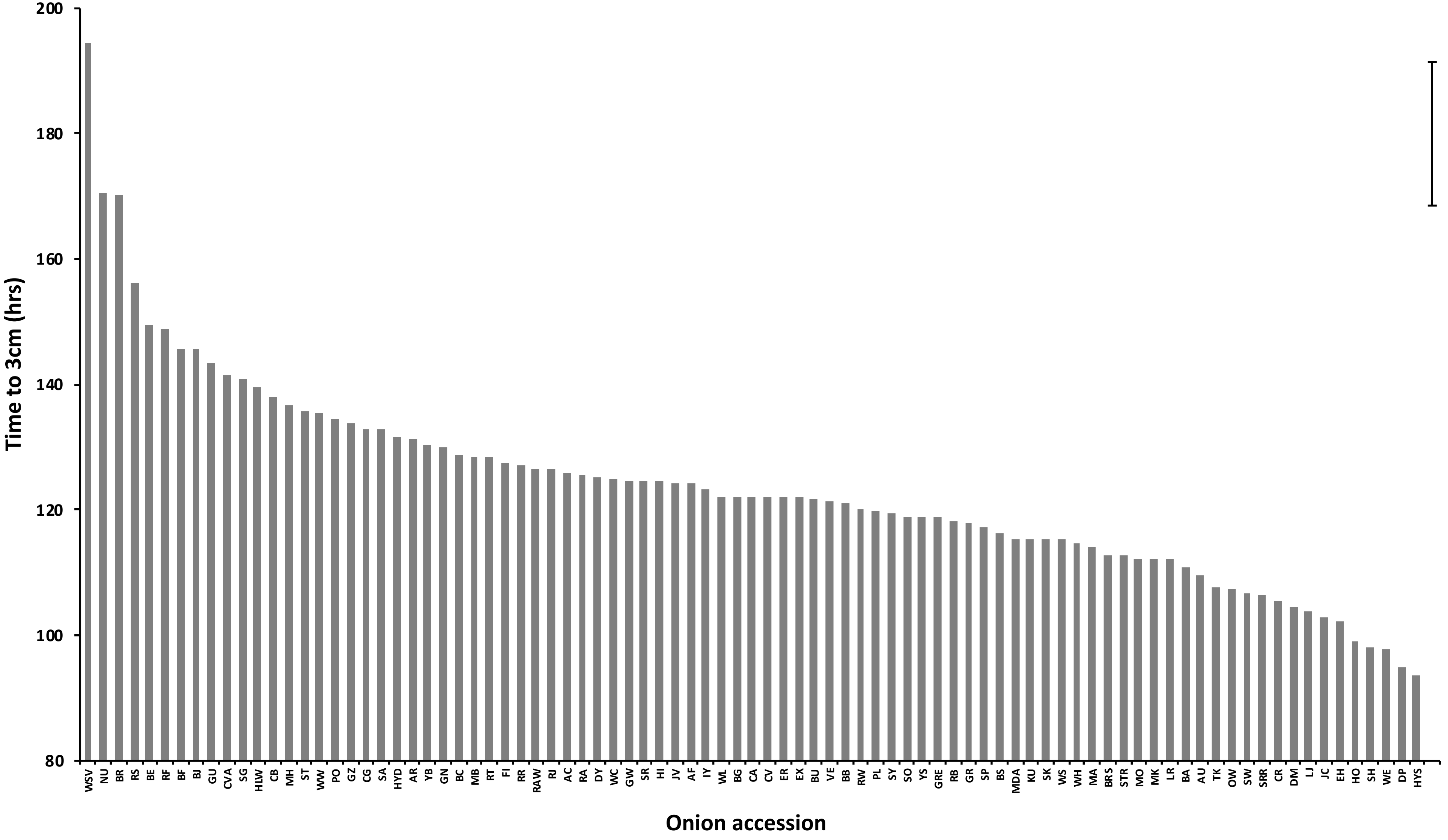

Fig. S2: Root growth rate of accessions in an onion diversity set as measured by time to reach 3cm in length. Error bar shows LSD (5% level).

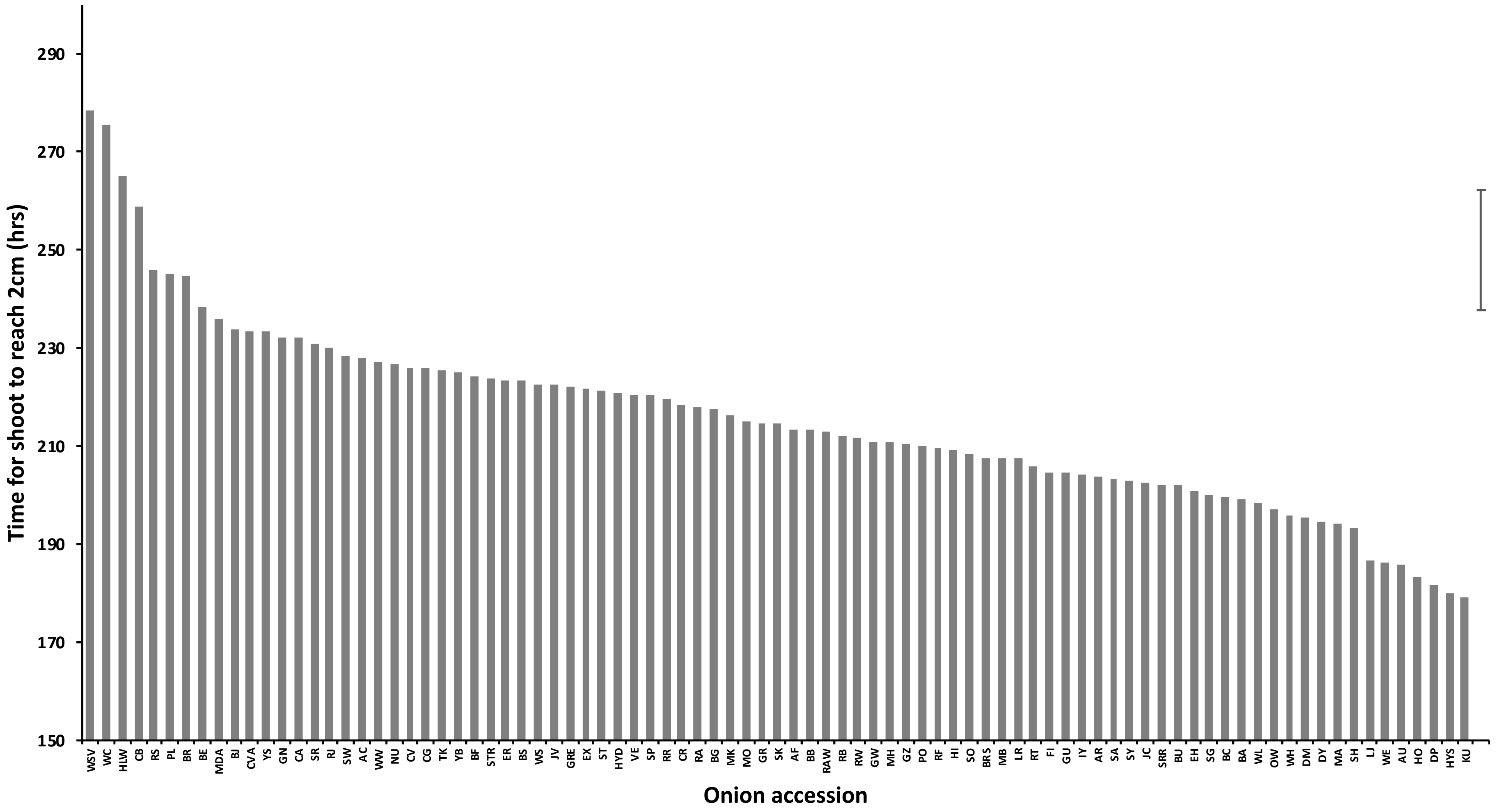

Fig. S3: Shoot growth rate of accessions in an onion diversity set as measured by time to reach 2cm in length. Error bar shows LSD (5% level).
