# Supplementary material for: Assembly and characterisation of a unique onion diversity set identifies resistance to Fusarium basal rot and improved seedling vigour
Source: Theor Appl Genet. 2019 Sep 13;132(12):3245–64. doi: 10.1007/s00122-019-03422-0 (PMC6820603; doi:10.1007/s00122-019-03422-0)
Supplement: Supplementary file 3 — Supplementary file3 (DOCX 168 kb) [file 122_2019_3422_MOESM3_ESM.docx]

**Assembly and characterisation of a unique onion diversity set identifies resistance to Fusarium basal rot and improved seedling vigour**

Theoretical and Applied Genetics

Taylor A*, Teakle GR, Walley PG, Finch-Savage WS, Jackson AC, Jones JE, Hand P, Thomas B, Havey MJ, Pink DAC, Clarkson JP.

*Warwick Crop Centre, School of Life Sciences, University of Warwick, andrew.taylor@warwick.ac.uk

**Table S3**: Percentage heterozygosity of half-sib parent accessions and half-sib individuals for four onion diversity set HS families. Up to ten half-sib parent lines and half-sib individuals were genotyped and heterozygosity calculated based on the 568 KASP^TM^ markers used in Fig 3.

| **Accession** | **Plant** | **Label** | **Heterozygocity %** |
| --- | --- | --- | --- |
| CA | seed from P1_7 | HS1_1 | 26.5 |
| CA | seed from P1_7 | HS1_2 | 24.3 |
| CA | seed from P1_7 | HS1_3 | 29.4 |
| CA | seed from P1_7 | HS1_4 | 18.1 |
| CA | seed from P1_7 | HS1_5 | 15.7 |
| CA | seed from P1_7 | HS1_6 | 31.6 |
| CA | seed from P1_7 | HS1_7 | 13.9 |
| CA | seed from P1_7 | HS1_8 | 28.6 |
| CA | seed from P1_7 | HS1_9 | 17.2 |
| CA | seed from P1_7 | HS1_10 | 23.5 |
| CA | Half sib mother plant | P1_1 | 37.6 |
| CA | Half sib mother plant | P1_2 | 37.8 |
| CA | Half sib mother plant | P1_3 | 37.3 |
| CA | Half sib mother plant | P1_4 | 37.2 |
| CA | Half sib mother plant | P1_5 | 37.2 |
| CA | Half sib mother plant | P1_6 | 40.8 |
| CA | Half sib mother plant | **P1_7** | 35.3 |
| CA | Half sib mother plant | P1_8 | 35.0 |
| CA | Half sib mother plant | P1_9 | 36.5 |
| CA | Half sib mother plant | P1_10 | 35.6 |
| DM | seed from P2_6 | HS2_1 | 3.0 |
| DM | seed from P2_6 | HS2_2 | 14.2 |
| DM | seed from P2_6 | HS2_3 | 15.7 |
| DM | seed from P2_6 | HS2_4 | 3.3 |
| DM | seed from P2_6 | HS2_5 | 13.1 |
| DM | seed from P2_6 | HS2_6 | 4.8 |
| DM | seed from P2_6 | HS2_7 | 4.2 |
| DM | seed from P2_6 | HS2_8 | 12.6 |
| DM | seed from P2_6 | HS2_9 | 3.9 |
| DM | seed from P2_6 | HS2_10 | 12.5 |
| DM | Half sib mother plant | P2_1 | 7.6 |
| DM | Half sib mother plant | P2_2 | 7.8 |
| DM | Half sib mother plant | P2_3 | 13.7 |
| DM | Half sib mother plant | P2_4 | 16.3 |
| DM | Half sib mother plant | P2_5 | 9.4 |
| DM | Half sib mother plant | **P2_6** | 6.2 |
| DM | Half sib mother plant | P2_7 | 13.6 |
| DM | Half sib mother plant | P2_8 | 14.7 |
| DM | Half sib mother plant | P2_9 | 14.0 |
| DM | Half sib mother plant | P2_10 | 5.6 |
| GR | seed from P3_7 | HS3_1 | 30.3 |
| GR | seed from P3_7 | HS3_2 | 26.7 |
| GR | seed from P3_7 | HS3_3 | 14.1 |
| GR | seed from P3_7 | HS3_4 | 30.2 |
| GR | seed from P3_7 | HS3_5 | 27.0 |
| GR | seed from P3_7 | HS3_6 | 11.6 |
| GR | seed from P3_7 | HS3_7 | 16.9 |
| GR | seed from P3_7 | HS3_8 | 13.8 |
| GR | seed from P3_7 | HS3_9 | 24.9 |
| GR | seed from P3_7 | HS3_10 | 28.7 |
| GR | Half sib mother plant | P3_1 | 30.5 |
| GR | Half sib mother plant | P3_2 | 29.4 |
| GR | Half sib mother plant | P3_3 | 20.3 |
| GR | Half sib mother plant | P3_4 | 31.3 |
| GR | Half sib mother plant | P3_5 | 28.5 |
| GR | Half sib mother plant | P3_6 | 30.5 |
| GR | Half sib mother plant | **P3_7** | 30.0 |
| GR | Half sib mother plant | P3_8 | 32.7 |
| GR | Half sib mother plant | P3_9 | 27.8 |
| GR | Half sib mother plant | P3_10 | 28.8 |
| HO | seed from P4_8 | HS4_1 | 9.4 |
| HO | seed from P4_8 | HS4_2 | 18.0 |
| HO | seed from P4_8 | HS4_3 | 31.8 |
| HO | seed from P4_8 | HS4_4 | 29.5 |
| HO | seed from P4_8 | HS4_5 | 29.0 |
| HO | seed from P4_8 | HS4_6 | 33.0 |
| HO | seed from P4_8 | HS4_7 | 34.1 |
| HO | seed from P4_8 | HS4_8 | 31.7 |
| HO | seed from P4_8 | HS4_9 | 19.2 |
| HO | seed from P4_8 | HS4_10 | 15.9 |
| HO | Half sib mother plant | P4_1 | 33.2 |
| HO | Half sib mother plant | P4_2 | 30.4 |
| HO | Half sib mother plant | P4_3 | 31.4 |
| HO | Half sib mother plant | P4_4 | 30.6 |
| HO | Half sib mother plant | P4_5 | 29.0 |
| HO | Half sib mother plant | P4_6 | 30.1 |
| HO | Half sib mother plant | **P4_8** | 37.3 |
| HO | Half sib mother plant | P4_9 | 35.2 |
| HO | Half sib mother plant | P4_10 | 32.0 |

**Table S4: Identification of the male parent of half-sib individuals**

1. Half-sib parent analysis for AcDFS accession CA
2. Half-sib parent analysis for AcDFS accession DM
3. Half-sib parent analysis for AcDFS accession GR
4. Half-sib parent analysis for AcDFS accession HO
5. P denotes a half-sib parent plant, HS denotes a half-sib plant, the female parent plant of the half-sib plants analysed here is indicated by Self. See Table S1 for the full details of each plant.
6. Total alleles refers to the number of alleles that were polymorphic, and therefore informative for each half-sib individual - half-sib parent comparison, out of the 892 KASP markers used to genotype these plants.
7. Conflicting alleles refers to the number of the polymorphic alleles that were incompatible with the half-sib parent individual being the male parent of the half-sib individual. For the genuine male parent there should be zero conflicting alleles.
8. Green highlighted values indicate the deduced male parent plant.

a)

b)

c)

d)
